# Genetic differentiation and signatures of local adaptation revealed by RADseq for a highly dispersive mud crab *Scylla olivacea* (Herbst, 1796) in the Sulu Sea

**DOI:** 10.1002/ece3.7625

**Published:** 2021-05-04

**Authors:** Michael John R. Mendiola, Rachel Ravago‐Gotanco

**Affiliations:** ^1^ The Marine Science Institute University of the Philippines Diliman Quezon City Philippines

**Keywords:** marine connectivity, mud crab, population genomics, RAD sequencing, seascape genetics, Sulu Sea

## Abstract

Connectivity of marine populations is shaped by complex interactions between biological and physical processes across the seascape. The influence of environmental features on the genetic structure of populations has key implications for the dynamics and persistence of populations, and an understanding of spatial scales and patterns of connectivity is crucial for management and conservation. This study employed a seascape genomics approach combining larval dispersal modeling and population genomic analysis using single nucleotide polymorphisms (SNPs) obtained from RADseq to examine environmental factors influencing patterns of genetic structure and connectivity for a highly dispersive mud crab *Scylla olivacea* (Herbst, 1796) in the Sulu Sea. Dispersal simulations reveal widespread but asymmetric larval dispersal influenced by persistent southward and westward surface circulation features in the Sulu Sea. Despite potential for widespread dispersal across the Sulu Sea, significant genetic differentiation was detected among eight populations based on 1,655 SNPs (*F_ST_* = 0.0057, *p* < .001) and a subset of 1,643 putatively neutral SNP markers (*F_ST_* = 0.0042, *p* < .001). Oceanography influences genetic structure, with redundancy analysis (RDA) indicating significant contribution of asymmetric ocean currents to neutral genetic variation ($R_{\text{adj}}^{2}$ = 0.133, *p* = .035). Genetic structure may also reflect demographic factors, with divergent populations characterized by low effective population sizes (*N*_e_ < 50). Pronounced latitudinal genetic structure was recovered for loci putatively under selection (*F_ST_* = 0.2390, *p* < .001), significantly correlated with sea surface temperature variabilities during peak spawning months for *S. olivacea* ($R_{\text{adj}}^{2}$ = 0.692–0.763; *p* < .050), suggesting putative signatures of selection and local adaptation to thermal clines. While oceanography and dispersal ability likely shape patterns of gene flow and genetic structure of *S. olivacea* across the Sulu Sea, the impacts of genetic drift and natural selection influenced by sea surface temperature also appear as likely drivers of population genetic structure. This study contributes to the growing body of literature documenting population genetic structure and local adaptation for highly dispersive marine species, and provides information useful for spatial management of the fishery resource.

## Graphical abstract

This research presents a seascape genomics approach using combined biophysical modeling and RADseq data to examine the genetic structure of a highly dispersive mud crab *Scylla olivacea* in the Sulu Sea, Philippines. We report significant genetic differentiation found using mostly neutral markers despite potential for widespread connectivity as indicated by the model simulations. Our study also highlights the pattern of latitudinal genetic structure as revealed by the outlier loci, suggesting local adaptation to environmental clines such as sea surface temperature.

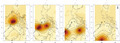

## 1 INTRODUCTION

Considering the spatial patterns and scales of dispersal, population connectivity has key implications for management and conservation (Moritz, 1994; Palumbi, 2003). For marine organisms, connectivity is primarily driven by complex interactions between life history characteristics and environmental conditions, which influence the dynamics and persistence of populations (Cowen & Sponaugle, 2009). The absence of apparent physical barriers in the ocean combined with high dispersal potential of most marine organisms shaped the paradigm of genetic homogeneity in the marine environment (Hauser & Carvalho, 2008). Advances in DNA sequencing technologies now provide adequate resolution to study population genetic processes through high‐throughput genotyping of single nucleotide polymorphisms (SNPs) using restriction site‐associated DNA (RAD) sequencing approaches (Andrews et al., 2016; Baird et al., 2008; Davey et al., 2011) such as ddRAD (Peterson et al., 2012), 2b‐RAD (Wang et al., 2012), and ezRAD (Toonen et al., 2013). Coupling genomic approaches with biophysical modeling, which simulates and tracks larval dispersal in the marine environment (reviewed in Swearer et al., 2019), has converged into a more integrative seascape genomics approach to examine processes that shape genetic variation, whether neutral or adaptive, in the marine environment (Riginos et al., 2016; Selkoe et al., 2016).

Seascape genomics studies have improved our understanding of the environmental conditions influencing population connectivity and the spatial distribution of genetic variation in the ocean. The ability to interrogate population diversity using thousands of SNPs, and to identify loci which may be under the influence of selection versus neutral loci (Davey et al., 2011; Gagnaire et al., 2015) enables examination of adaptive divergence in response to environmental factors. There is a growing body of literature documenting genetic structure either due to neutral variation or due to adaptation to environment, at finer spatial scales than expected from species dispersal potentials, among them: ocean currents (Benestan et al., 2016; Coscia et al., 2020; Dang et al., 2019; Gilg & Hilbish, 2003; Lal et al., 2017; Paterno et al., 2017; Riginos et al., 2019; Schunter et al., 2011; Teske et al., 2016; Truelove et al., 2017; Van Wyngaarden et al., 2018; Xuereb et al., 2018), temperature (Carreras et al., 2020; Chu et al., 2014; Coscia et al., 2020; Hoey & Pinsky, 2018; Sandoval‐Castillo et al., 2018; Wang et al., 2013), and salinity (Sjöqvist et al., 2015).

Mud crabs (genus: *Scylla* De Haan, 1833) are commercially important species with a wide distribution in mangrove areas throughout the Indo‐West Pacific and other tropical and subtropical regions (Alberts‐Hubatsch et al., 2015). Three mud crab species were identified in the Philippines (*S. olivacea, S. tranquebarica*, and *S. serrata*) following the description of Keenan et al. (1998), with *S. olivacea* (Herbst, 1796) being the most abundant (Lebata et al., 2007). While adult mud crabs exhibit limited movement (Hyland et al., 1984), larvae are thought to be highly dispersive due to their long pelagic larval duration (PLD) averaging 20–30 days (Ali et al., 2020; Jantrarotai et al., 2002; Motoh et al., 1977; Thirunavukkarasu et al., 2014), which can extend up to 75 days under suboptimal conditions of temperature and salinity (*S. serrata;* Baylon, 2010). Although ocean currents play a huge role in larval dispersal and settlement success (Cowen & Sponaugle, 2009), survival and development of mud crab larvae strongly depends on water temperature and salinity (Baylon, 2011; Hamasaki, 2003; Hill, 1974; Nurdiani & Zeng, 2007). In the Sulu Sea basin, *S. olivacea* populations are distributed along regions influenced by temporally varying environmental gradients (Oppo et al., 2003) and complex sea surface circulation such as the southward‐flowing Sulu Sea throughflow from the South China Sea, the westward‐flowing Bohol Sea current exiting via the Dipolog Strait, and the southern Sulu Sea gyre (Han et al., 2009; Hurlburt et al., 2011). Oceanographic features in the Sulu Sea have been suggested to act as barriers to gene flow among populations for other taxa with relatively lower dispersal potentials than *S. olivacea* such as the seahorse *Hippocampus spinosissimus* (Lourie et al., 2005), damselfish *Dascyllus aruanus* (Raynal et al., 2014), and sea cucumber *Holothuria scabra* (Ravago‐Gotanco & Kim, 2019). There is limited information, however, on the genetic structure of Philippine populations of *S. olivacea,* with one study reporting weak but significant genetic differentiation of Philippine populations based on microsatellite loci (Paran & Ravago‐Gotanco, 2017). Moreover, there are no studies to date that explicitly examined the influence of asymmetric ocean currents and environmental heterogeneity on population connectivity and genetic structure of a highly dispersive species in the Sulu Sea.

This study examined patterns of connectivity among populations of the orange mud crab (*S. olivacea*) in the Sulu Sea basin. Using a seascape genomics approach, we aimed to: (a) characterize genetic structure of *S. olivacea* across the Sulu Sea and examine spatial patterns of genetic connectivity using SNP markers generated by RADseq; (b) examine the influence of oceanographic circulation on genetic structure and connectivity of *S. olivacea* populations in the Sulu Sea; and (c) examine the SNP dataset for signatures of local adaptation, which may be correlated with other environmental factors. First, we developed a biophysical model of larval dispersal parameterized using the life history characteristics of *S. olivacea*, to generate realistic predictions of larval dispersal and connectivity in the Sulu Sea. We combined larval dispersal estimates with empirical genetic observations at neutral loci to determine the influence of asymmetric ocean currents on spatial patterns of connectivity. Second, we performed analyses to recover loci putatively under selection to examine signatures of local adaptation. We assessed the potential impact of environmental factors, specifically sea surface temperature and rainfall (as a proxy for salinity) on adaptive divergence of *S. olivacea*. The results of this study provide valuable insights into the spatial scales of dispersal, patterns of genetic structure, and the influence of environmental and evolutionary processes on population connectivity of *S. olivacea* in the Sulu Sea basin, to support the development of management and conservation strategies for the fishery resource.

## 2 MATERIALS AND METHODS

### 2.1 Larval dispersal simulation in the Sulu Sea basin

A larval dispersal model was developed to examine connectivity of *S. olivacea* in the Sulu Sea. Seven larval release sites were designated, coinciding with locations where samples were collected for genetic analysis, with the exception of Coron (Table 1). Larval dispersal simulations were performed using the connectivity modeling system (CMS; Paris et al., 2013). The CMS is a model that couples Lagrangian‐based descriptions of ocean circulation with individual‐based modeling to simulate the movement of particles each with individual behaviors parameterized from known biological traits, for example, larval duration, mortality, and settlement behavior. The model was configured using contemporary oceanographic data from the 3D Global Hybrid Coordinate Ocean Model (HYCOM; Chassignet et al., 2007) with 1/25° (~4.4 km) horizontal resolution, simulating realistic sea surface (0−10m) currents throughout the Sulu Sea. One year of HYCOM outputs from March 2015 to February 2016 was used to run the model, covering the reversing monsoon wind forcing in the region (Han et al., 2009) and the year‐round spawning season of *Scylla* in the Philippines (Arriola, 1940). A basin‐scale habitat map was included in the model using the Philippine mangrove Landsat data of Long and Giri (2011), generating 159 larval settlement nodes along the boundaries of the basin. Particle release site coordinates were adjusted up to 12 km away from the coast to simulate the offshore spawning migration reported for *S. olivacea* (Koolkalya et al., 2006; Moser et al., 2005). The model was configured to release fifty thousand particles from each release site weekly over a period of 1 year, for a total of 18.5 million larval particles released from seven source nodes. Released particles were parameterized as competent to settle after 20 days of passive dispersal, with a maximum duration in the water column up to 30 days based on the pelagic larval duration of *S. olivacea* inferred from observations on the timing of peak spawning and recruitment of juveniles to estuaries (Ali et al., 2020). To account for larval mortality, the number of particles was set to be reduced by half after 4 days of release based on the reported mortality of *Scylla* from larval stages zoea I to III (Jantrarotai et al., 2002; Thirunavukkarasu et al., 2014). The resulting probability estimates of larval dispersal (measured as percent settlement) were postprocessed to generate a population‐by‐population connectivity matrix. This was done by assigning settlement nodes to their respective islands or nearby sampling locality (~75 km radius), resulting in a reduced population source–sink dataset.

**TABLE 1:** Summary information of *Scylla olivacea* sample location and genetic diversity estimates

Sampling location	Site code	Group	*N*	Sampling Coordinates Lat, Long	Adjusted Coordinates Lat, Long	*H_O_*	*H_E_*	*F_IS_*
Coron, Palawan	CRN	West	14	12.156, 120.094	–, –	0.276	0.275	−0.001
Roxas, Palawan	ROX	West	13	10.364, 119.389	10.296, 119.416	0.242	0.269	0.098
Puerto Princesa, Palawan	PPC	West	15	9.7384, 118.687	9.700, 118.783	0.231	0.282	0.181
Bataraza, Palawan	BAT	West	12	8.650, 117.520	8.628, 117.557	0.240	0.266	0.096
San Jose, Occidental Mindoro	MSJ	East	22	12.346, 121.063	12.307, 121.012	0.250	0.275	0.094
Hamtic, Antique	ANT	East	13	10.719, 121.964	10.690, 121.925	0.263	0.277	0.050
Siaton, Negros Oriental	NEG	East	15	9.048, 123.002	9.026, 122.997	0.256	0.273	0.062
Languyan, Tawi‐Tawi	TWI	East	12	5.215, 119.955	5.252, 119.862	0.245	0.267	0.080
Sta. Ana, Cagayan	CGY	Outgroup	15	18.480, 122.150		0.246	0.273	0.098
General Santos City	GSC	Outgroup	15	6.105, 125.167		0.223	0.278	0.197

Note

*N* = Number of individuals; *H_O_* = observed heterozygosity; *H_E_* = expected heterozygosity; *F_IS_* = inbreeding coefficient.

### 2.2 Sample collection, species identification, and total DNA extraction

Adult *Scylla olivacea* (*n* = 146) were collected from natural mangrove habitats from 8 sites in the Sulu Sea and 2 outlier locations between 2016 and 2017 (Table 1). Species were identified using morphological characters following the description of Keenan et al. (1998). Tissue samples were obtained from mud crab pereopods, preserved in salt‐saturated DMSO‐EDTA (SSDE) solution (Dawson et al., 1998), and stored at room temperature until analysis. Specimens were also identified using species diagnostic molecular markers following the protocol of Ma et al. (2012). DNA was extracted using the GeneJET Genomic DNA Purification Kit (Thermo Scientific), following the manufacturer's instructions with some modifications. DNA concentration was quantified using a Qubit^®^ fluorometer. DNA quality was assessed by agarose gel electrophoresis and measurement of absorbance ratios (A260/280) using a NanoDrop™ spectrophotometer.

### 2.3 Double‐digest RAD (ddRAD) sequencing

Double‐digest restriction site‐associated (ddRAD) libraries were prepared according to Peterson et al. (2012). DNA extracts were run on an agarose gel to check for DNA quality. Samples with low molecular weight smears were further purified using paramagnetic beads (SPRIselect; Beckman Coulter). Approximately 150 ng DNA from individual samples was digested with *MluCI* and *MspI* and purified using AMPure XP (Beckman Coulter). Custom‐barcoded adapters P1 and P2 (see Peterson et al. (2012) for sequences) were then ligated to ~50 ng of DNA. The P1 adapter includes a 5bp inline unique sequence for individual barcoding. Groups of 48 samples with unique barcodes were pooled (equal volumes of each sample), purified, and size‐selected using a BluePippin system (target insert size of 400–500 bp). Unique external indices were added to each pool by PCR amplification. PCR products were purified, fragment sizes were verified, qPCR was quantified, and PCR products were further pooled in equimolar quantities. Sequencing of DNA libraries was performed using the Illumina NovaSeq™ 6,000 Sequencing System with S4 flow cell type. Library construction and sequencing was performed at the Genomics Core Lab, Texas A&M University, Corpus Christi.

### 2.4 Read processing and SNP filtering

Sequence libraries were initially demultiplexed using the internal barcodes, while reads with low‐quality scores, uncalled bases, and sequences with intact adapters were removed using the module *process_radtags* in STACKs v2.2 (Rochette et al., 2019). Raw read quality scores (Phred33) and adapter contamination were examined through FastQC v0.10.1 (Andrews, 2010) and MultiQC v1.7 (Ewels et al., 2016). The STACKs pipeline module *denovo_map.pl* was used for the construction of stacks and generation of initial catalog of putative SNPs. Stack assembly parameters such as the minimum depth of coverage required to create a stack (−*m*) was set to 5 (default: 3), and the maximum distance (in nucleotides) allowed between stacks (−*M*) and the number of mismatches allowed between sample tags when generating the catalog (−*n*) were increased to 4 (default: 3) to increase the SNP calling confidence and to minimize missing data (Lal et al., 2016). Additional filtering steps were performed in the *populations* module with the following criteria: retain only the first SNP per locus, locus must be present in all populations (−*p* = 10), and exclude loci that were not present in at least 50% of the individuals for each population (−*r* = 0.5).

Postprocessing of the SNP panel was done to exclude SNPs that were not genotyped in at least 70% of the individuals across the entire dataset. Loci with up to 30% missing data were excluded using *poppr* v2.8.3 (Kamvar et al., 2014), to minimize the effect of missing data on population structure inference (Reeves et al., 2016). SNP markers with minor allele frequencies (MAFs) less than 0.05 across all sites were excluded, to eliminate loci with lower power to detect genetic variability (Ardlie et al., 2002; DeWoody & DeWoody, 2005). The Hardy–Weinberg equilibrium (HWE) tests at the population level were conducted using the package *pegas* v0.11 (Paradis, 2010). Loci that exhibited significant deviation from HWE (false discovery rate (FDR)‐adjusted *p* < .05) in at least 50% the populations (5 or more populations) were excluded. To limit the influence of nonindependent loci, linkage disequilibrium (LD) was tested between all SNP pairs using the package *genetics* v1.3.8.1.2 (Warnes et al., 2019), and SNPs at strong linkage disequilibrium (*r* ^2^ > 0.8) were removed (Lee et al., 2018; Tian et al., 2009). SNPs with high observed heterozygosity (*H_o_* > 0.6) were also dropped from the dataset (e.g., Ackiss et al., 2018; Hohenlohe et al., 2010; Van Wyngaarden et al., 2017) using *pegas* v0.11 (Paradis, 2010), to eliminate loci exhibiting extremely high heterozygosity resulting from false SNP calls or assembly errors (Lee et al., 2018). Analyses were performed in R version 3.5.3 (R Core Team, 2019).

### 2.5 Identifying non‐neutral SNPs

Loci were identified as being putatively neutral or under selection using three differentiation‐based (*F_ST_*) outlier detection methods, which use different underlying models: BayeScan v2.1 (Foll & Gaggiotti, 2008), Arlequin v3.5.2.2 (Excoffier & Lischer, 2010), and OutFLANK v0.2 (Whitlock & Lotterhos, 2015). BayeScan uses a Bayesian likelihood approach to estimate the posterior probability that a locus is under selection under the assumption that allele frequencies within populations follow the multinomial–Dirichlet distribution (Feng et al., 2015; Foll & Gaggiotti, 2008). We performed the analyses on 20 pilot runs with 100,000 iterations and a burn‐in of 50,000 steps. SNPs were then identified as outliers using a false discovery rate (FDR) *q*‐value threshold of 0.05. Arlequin uses a hierarchical island model, which compares observed locus‐specific *F_ST_* to the observed global *F_ST_* value using coalescent simulations (Excoffier & Lischer, 2010). We performed a total of 20,000 simulations consisting of 10 simulated groups with 100 demes per group to detect loci under selection. SNP markers with significant *F_ST_* values at the 99% confidence interval (CI) limit were considered as putative outlier loci. OutFLANK uses a maximum‐likelihood approach to generate the *F_ST_* distribution of neutral loci by trimming extreme values (Whitlock & Lotterhos, 2015). We ran OutFLANK with the recommended parameters: left and right trimming fraction of 0.05, minimum heterozygosity of 0.1, and identified outlier loci using an FDR q‐value threshold of 0.05. To reduce the number of false positives and adopt a conservative approach to identifying putative outlier loci, only loci identified by at least two tools were considered as outliers.

Based on the results of outlier loci analyses, we generated three SNP datasets consisting of: (a) all loci; (b) putatively neutral loci; and (c) putative outlier loci only, hereafter referred to as all, neutral, and outlier loci, respectively. For putative function annotation, consensus tags associated with candidate outlier loci were queried for sequence similarity against the NCBI nucleotide (nr/nt) collection using the BLAST algorithm BLASTN v2.6.1 (Morgulis et al., 2008; Zhang et al., 2000), with an expected threshold of 0.05.

### 2.6 Population genetic structure and effective population size

The three SNP panels (all, neutral, and outlier loci) were used to examine genetic differentiation and infer connectivity among populations. We used Weir and Cockerham's *F_ST_* (1984) to estimate genetic differentiation over all populations (global *F_ST_*) and between populations (pairwise *F_ST_*), calculated using the R packages *hierfstat* v0.04‐22 (Goudet & Jombart, 2019) and *dartR* v1.1.11 (Gruber et al., 2018), respectively. Significance of *F_ST_* values was tested with 10,000 bootstrap replicates, and *p*‐values for population pairwise comparisons were adjusted for multiple tests using FDR. Pairwise *F_ST_* values were visualized using heatmaps with dendrograms generated from hierarchical clustering analysis performed using the function *heatmap.2* from the *gplots* v3.1.0 package for R (Warnes et al., 2020).

Spatial patterns of genetic structure were examined using a discriminant analysis of principal components (DAPC; Jombart et al., 2010) implemented in the R package *adegenet* v2.1.3 (Jombart, 2008). DAPC was performed including the sampling location of each individual as prior information. The number of principal components to retain for DAPC was chosen following cross‐validation using the function *xval.DAPC* with 100 replicates to avoid issues of overfitting (Jombart et al., 2010). We also used GENELAND v4.0.8 (Guillot et al., 2005), a package that incorporates geographic coordinate information to estimate the number of genetic clusters and infer genetic landscapes across the Sulu Sea. We used the correlated allele frequency model with the following recommended parameters: Number of possible clusters (K) that were initially set from 1 to 10 with 100,000 Markov chain Monte Carlo (MCMC) iterations, thinning of 100, burn‐in of 200, maximum rate of the Poisson process fixed to 146 (*N* = number of individuals), maximum number of nuclei in the Poisson–Voronoi tessellation process fixed to 438 (N multiplied by 3), and individual samples from each location that were set to the same spatial coordinates. Postprocessing of MCMC outputs generated a final estimate of K, which was used as the maximum number of populations in succeeding runs performed with 10 independent replicates. Individual runs were ranked, and the run having the highest average posterior probability was used to calculate individual membership coefficients and maps of posterior probability of membership in each K cluster.

Hierarchical analysis of molecular variance (AMOVA; Excoffier et al., 1992) was performed to test for population structure inferred from *F_ST_* analysis and DAPC on the three SNP datasets. AMOVA was performed using *poppr* v2.8.3 (Kamvar et al., 2014), with significance tested using 1,000 permutations. We examined the putatively neutral dataset for patterns of isolation by distance using the *gl.ibd* function in *dartR*, which performs a Mantel test (number of permutations = 9,999) to assess correlation between log(geographic distance) and linearized genetic distance (*F_ST_*/(1 − *F_ST_*)) matrices. Geographic distances were measured as the shortest distance over water between all pairs of sites in the Sulu Sea using the *igraph* package for R (Csardi & Nepusz, 2006). Effective population size was estimated for each population based on putatively neutral loci only, using the linkage disequilibrium method (*N_eLD_*) of NeEstimator v2.01 (Do et al., 2014).

### 2.7 Gene flow estimates

Migration rates and the direction of gene flow between populations at the eastern and western boundaries of the Sulu Sea were estimated using the Bayesian strategy implemented in Migrate‐n v4.4.4 (Beerli & Felsenstein, 2001). To make the analyses computationally tractable, we analyzed only two populations, generated by pooling eastern sites MSJ, ANT, and NEG, into a single population (East), and western sites ROX and BAT into a single population (West) on the basis of apparent panmixia (pairwise *F_ST_ p* > .05). A randomly generated subset of 1,000 loci was used to calculate mutation‐scaled migration rates (*M* = *m*/μ, where *m* = immigration rate per generation and *μ* = mutation rate per site) and examine the probabilities of four different migration models: (a) a single genetic population (panmixia); (b) bidirectional gene flow between East and West allowing for asymmetric migration rates; (c) unidirectional gene flow from East to West; and (d) unidirectional gene flow from West to East. Analyses were performed on the CIPRES Portal (Miller et al., 2010). Initial runs starting with prior parameters for population size (*θ* = 0.1) and the migration rate (*M* = 2000) exhibited acceptable posterior distributions and were used for subsequent analyses. Each model was run using Metropolis sampling and a static heating scheme with four chains, 1 × 10^6^ generations with 10 replicate chains sampling every 100 steps, and a burn‐in of 100,000 steps. To determine the most likely migration model, log Bayes factors (LBFs) were calculated from the Bezier approximation score and compared for all four gene flow models (Beerli & Palczewski, 2010).

The directionality and rates of contemporary migration were examined using the Web application of *divMigrate* (https://popgen.shinyapps.io/divMigrate‐online/), which calculates the relative levels of migration by assessing the genetic differentiation between two populations and a hypothetical pool of migrants (Sundqvist et al., 2016). We used Nei's *G_ST_* estimated from neutral loci. The eight Sulu Sea populations were pooled into 5 groups, where sites were pooled in the absence of significant differentiation based on *F_ST_* values (see results). This pooling scheme is similar to the groupings used for the Migrate‐n analysis with one group of East sites (MSJ, ANT, and NEG) and one group of pooled West sites (ROX and BAT), with three additional populations: PPC, CRN, and TWI.

### 2.8 Genetic structure and environmental factors

To examine the influence of environmental variables on genetic structure of *S. olivacea* in the Sulu Sea basin, we used redundancy analysis (RDA), a direct gradient analysis technique, to test for significant relationships between response and explanatory variables (Legendre et al., 2011). The Hellinger‐transformed allele frequencies (Legendre & Gallagher, 2001) from neutral and outlier SNP datasets were used as the response variable, with the following environmental factors as the explanatory variables: directional ocean currents, sea surface temperature (SST), and rainfall.

The effect of ocean currents on genetic connectivity estimated from neutral loci was assessed by first transforming particle dispersal estimates derived from biophysical modeling into a set of synthetic variables known as asymmetric eigenvector maps (AEMs; Blanchet et al., 2008) from which predicted spatial patterns of genetic connectivity were generated (Blanchette et al., 2008; Riginos et al., 2019; Xuereb et al., 2018). AEM eigenfunctions were generated by creating a site‐by‐edge (binary) matrix of connections between all pairs of sites. Weight was then attributed to each edge, and when connectivity between a given pair of sites was greater than 0 in both directions, only the direction with the highest probability of dispersal was retained. AEM eigenfunctions were generated using *adespatial* v0.3‐7 (Dray et al., 2020) in R v3.5.3. The contributions of individual AEMs on neutral genetic variation were then calculated using redundancy analysis.

The influence of environmental factors such as SST and rainfall variabilities on adaptive genetic variation (local adaptation) was evaluated through redundancy analysis using the outlier dataset. For SST, high‐resolution (0.25° × 0.25°) monthly variability data (SST_mean_, SST_min_, and SST_max_) for the Sulu Sea domain (5–14°N, 116–124°E) from 1987 to 2005 were generated from the NOAA/OAR/ESRL PSL website at https://psl.noaa.gov/ (Reynolds et al., 2007). Similarly, fine‐scale rainfall data (mm/day) covering the Sulu Sea basin from 1998 to 2014 were obtained from the Tropical Rainfall Measuring Mission (TRMM) database (Huffman et al., 2007). SST and rainfall data were extracted for latitudes covering the range where samples for genetic analysis were collected.

All explanatory variables (AEMs, SST, and rainfall) were individually tested using a backward selection procedure with 999 permutations using *ordistep* in *vegan* v2.5‐6 (Oksanen et al., 2019), to retain the most important explanatory variables. Selected variables were included in the model, and the adjusted coefficient of determination ($R_{\text{adj}}^{2}$) was calculated. Partial RDA was performed, and the global analysis of variance was determined using the *ANOVA* function in *stats v3.5.3* with 999 permutations. The significance of individual RDA axes was also assessed using *ANOVA* (permutations = 999), and selected environmental vectors were fitted into the ordination using *envfit* (permutations = 999).

## 3 RESULTS

### 3.1 Larval dispersal estimates

**FIGURE 1:**
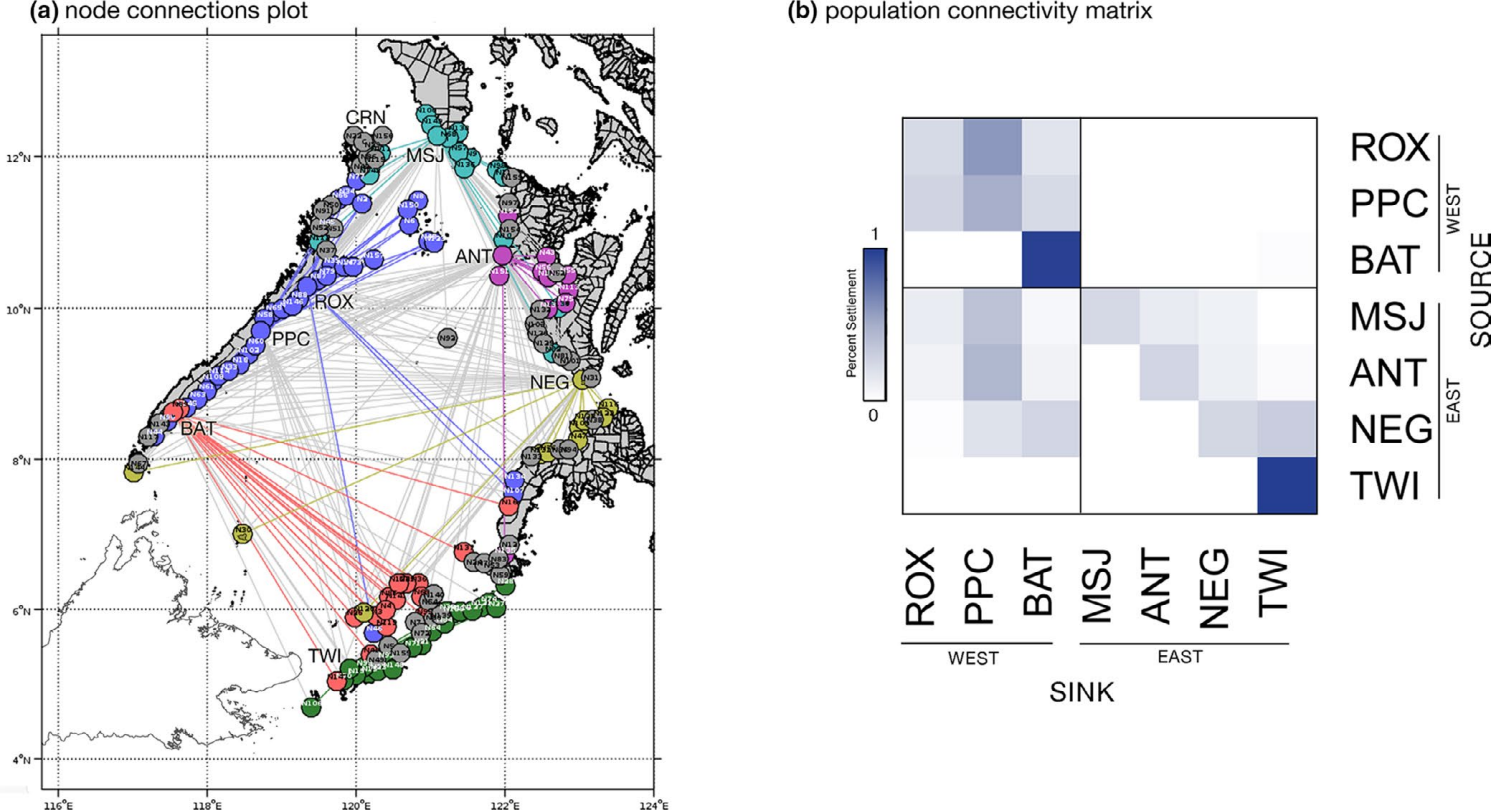
Estimated larval dispersal and connectivity of *Scylla olivacea* populations in the Sulu Sea basin from Lagrangian simulations. (a) Node connections plot generated from the larval dispersal estimates resulted in six population clusters indicated by the colored nodes and connections, gray links indicating connections between clusters. Site codes represent particle release sites, and settlement nodes are labeled numerically. (b) Population connectivity matrix showing the proportion of larvae successfully settled from the source locations (*y*‐axis) to settlement areas (*x*‐axis). Sulu Sea populations were segregated according to their boundary location (east or west). See Table 1 for location codes

Lagrangian simulations of larval dispersal using a parameterized biophysical model demonstrate ocean current‐mediated connectivity among *Scylla olivacea* populations in the Sulu Sea basin (Figure 1a). Predicted particle settlement patterns indicate predominantly southward dispersal along the western boundary. A greater proportion of particles released from ROX settled in PPC (60.1%) compared with PPC particles dispersing northward and settling in ROX (27.3%) (Figure 1b, Table S1) predominantly. Particles from ROX and PPC settled in BAT (39.3%). Conversely, very low proportions of particles released from BAT were predicted to settle northward to ROX and PPC (0.004%). A small proportion of larvae released from BAT were transported to TWI (1.8%), and the majority of BAT particles were self‐recruited (97.8%). Similar patterns of greater southward dispersal are observed along the eastern boundary populations (Figure 1b), although the predicted levels of particle connectivity were lower compared with western boundary populations. Particles released from MSJ were predicted to settle in ANT (12.70%) and NEG (9.21%), particles from ANT settled in NEG (10.4%) and TWI (2.70%), and particles from NEG settled in TWI (31.06%).

Across the Sulu Sea, dispersal simulations reveal a clear pattern of westward dispersal, with larval particles released from eastern boundary sites settling in western boundary sites (Figure 1b). In particular, particles from MSJ and ANT settled on three eastern sites: PPC (37.7% and 45.4%), ROX (11.7% and 8.10%), and BAT (5.00% and 7.80%). Particles released from NEG settled in PPC (17.6%) and BAT (26.2%). Self‐recruitment was relatively higher for PPC (49.7%), while TWI exhibited complete self‐recruitment (100%). There was no predicted settlement of particles released from western boundary sites to eastern sites. The larval dispersal model clearly reveals asymmetric transport across the Sulu Sea basin, with larval dispersal predominantly southward, and from eastern to western boundary sites.

### 3.2 SNP filtering and identification of non‐neutral loci

A total of 661,372,129 paired‐end (PE) reads from 146 individual *S. olivacea* libraries were processed for SNP discovery and filtering using STACKs v2.2. Out of 777,762 loci, 491,192 (63.2%) were aligned with PE contigs, with an effective per‐sample mean coverage of 20.8×. Following successive filtering steps (detailed in Table 2), a final dataset of 1,655 high‐quality, polymorphic SNP markers was recovered. BayeScan identified 12 putative outlier loci based on posterior probabilities at the 95% Bayes factor threshold, with *F_ST_* values ranging from 0.0919 to 0.5308 and positive alpha values (range = 2.02–4.67) suggestive of diversifying selection (Foll & Gaggiotti, 2008). Arlequin identified 87 putative outlier loci (*F_ST_ p*‐values less than .0099) at the 99% confidence interval limit, which also included the 12 loci identified by BayeScan as putative outliers. OutFLANK flagged 12 loci as putative outliers (*F_ST_* = 0.1124–0.3828); of these, 8 loci were concordant with BayeScan and Arlequin analyses. Consequently, a SNP panel consisting of 12 candidate outlier loci identified by at least two outlier tests was designated as the outlier loci dataset. Querying the contig sequences of putative outlier loci against public domain sequences using a BLAST showed no functional gene region matches for these 12 outlier loci (Table S2).

**TABLE 2:** Filtering processes and specific parameters used to filter the final SNP panel of *Scylla olivacea*

Filtering steps	No. of removed loci	No. of retained loci
STACKs (*denovo_map.pl*)		
−*m* 5		
−*M* 4		
−*n* 4	–	777,762
STACKs (*populations*)	762,394	15,368
−*r* 0.50		
−*p* 10		
Loci with >30% missing data	513	14,855
MAF <0.05, informative loci	12,211	2,644
HWE per population	9	2,635
LD (*r* ^2^ > 0.8)	970	1,665
Observed heterozygosity (>0.6)	10	1,655
Final SNP panels		1,655

Note

Each filtering step indicates the number of SNPs that were removed and retained. Filtering tools and programs are indicated in Methods section.

### 3.3 Genetic differentiation and population structure

**FIGURE 2:**
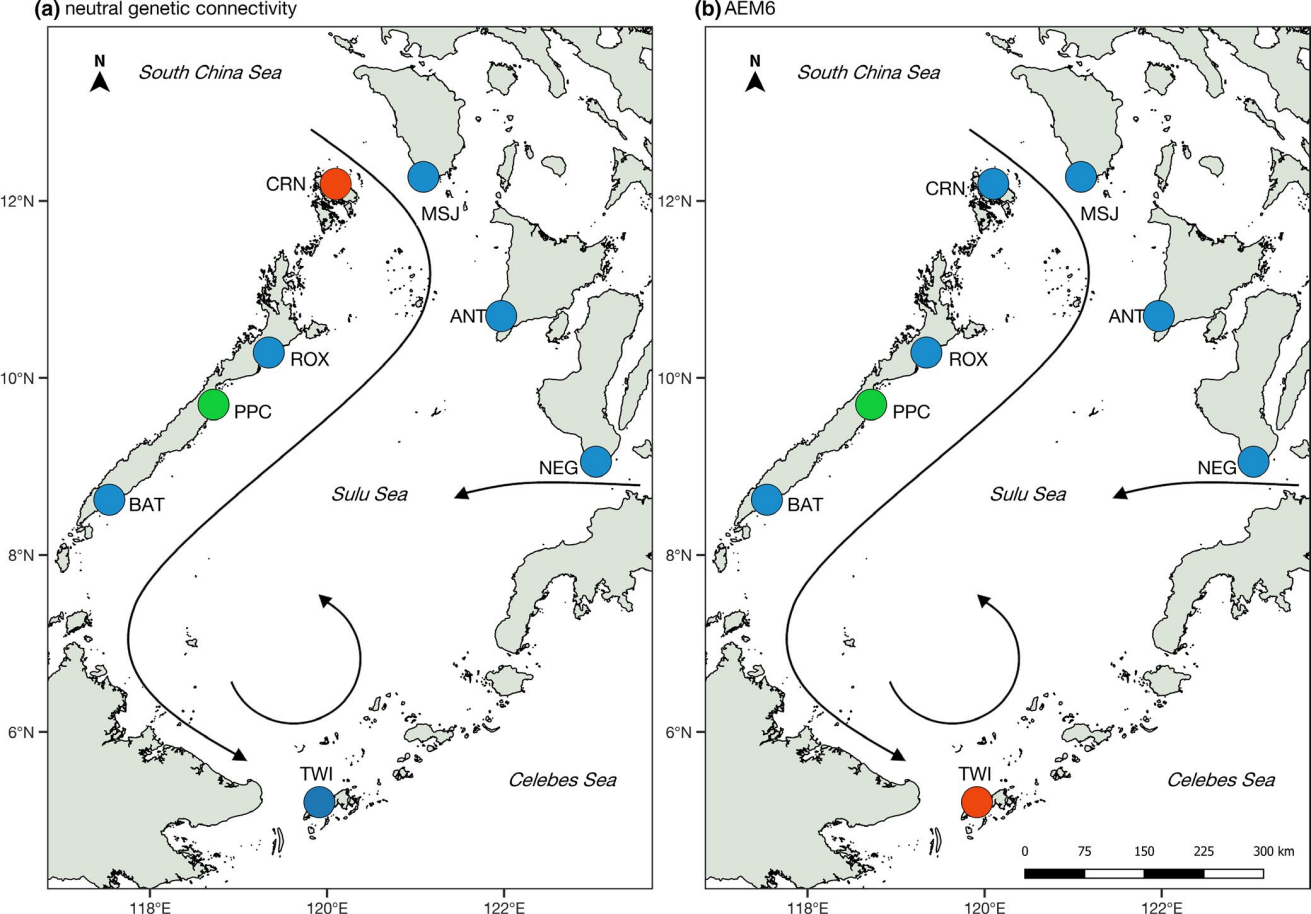
Spatial patterns of *Scylla olivacea* connectivity based on (a) empirical genetic estimates (pairwise *F_ST_*) from the putatively neutral loci (1,643 SNPs); and (b) asymmetric larval dispersal estimates (AEM6) from the particle dispersal simulations in the Sulu Sea. Sampling points are colored according to their cluster assignment. Persistent surface currents in the Sulu Sea are shown

**FIGURE 3:**
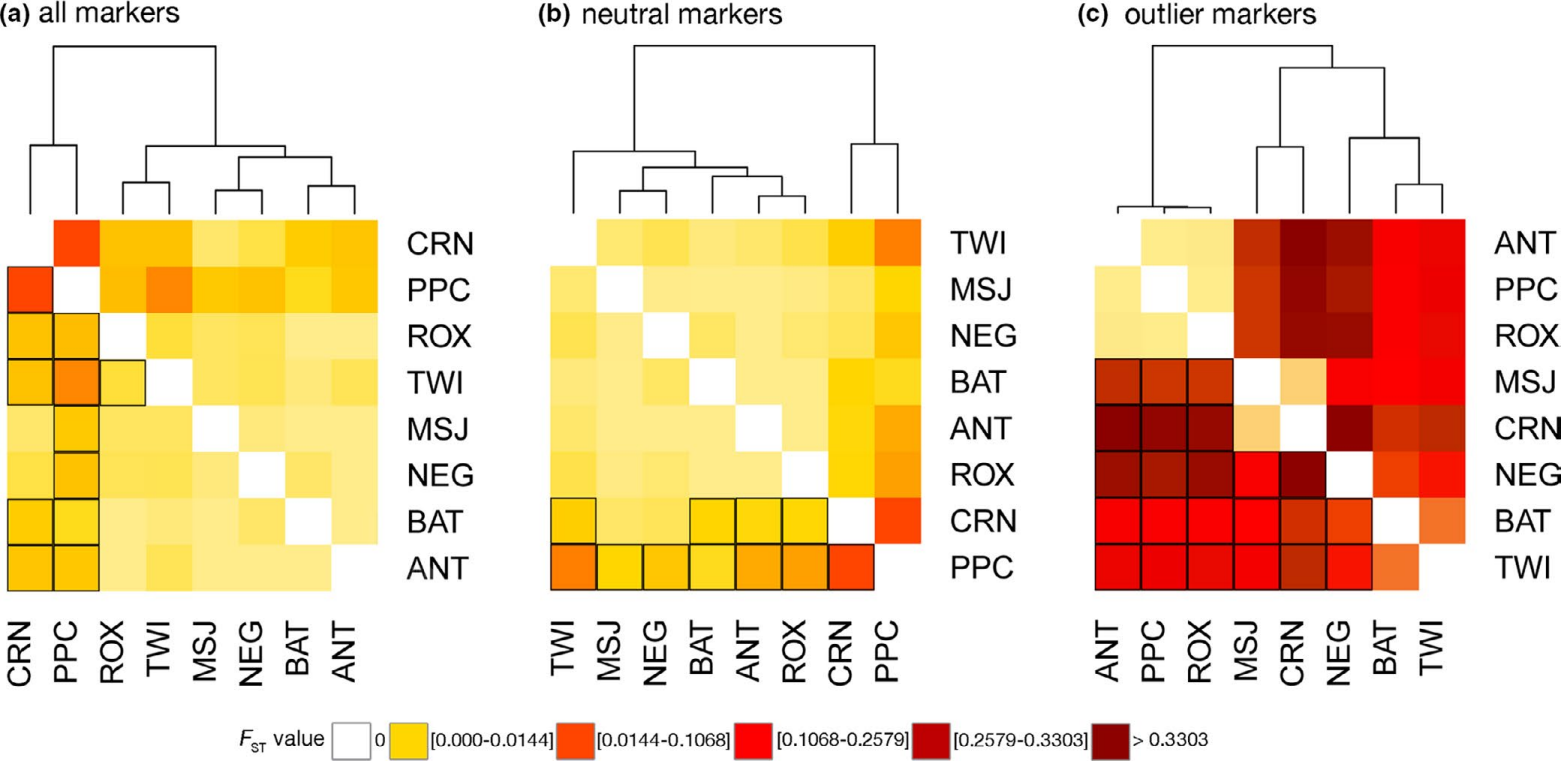
Heatmap of pairwise genetic differentiation (*F_ST_*) of *Scylla olivacea* between sampling locations in the Sulu Sea, based on Weir and Cockerham's weighted estimates using (a) all markers (1,655 loci), (b) neutral‐only markers (1,643 SNPs), and (c) outlier only (12 SNPs). All points were clustered by pairwise *F_ST_* values, based on classification and hierarchical clustering method. Site combinations (below diagonal) with boxed bold lines indicate significant genetic differentiation following FDR correction for multiple tests (*p* < .05)

*Scylla olivacea* populations exhibited significant genetic differentiation based on global estimates of *F_ST_* calculated using all loci (1655 SNPs; *F_ST_* = 0.0070, *p* = .001) and putatively neutral loci (1643 SNPs; *F_ST_* = 0.0056, *p* = .001). Excluding outgroup sites, Sulu Sea samples (*n* = 116 individuals; 8 sites) still exhibited significant genetic differentiation for all loci (*F_ST_* = 0.0057, *p* = .001) and neutral loci (*F_ST_* = 0.0042, *p* = .001; Figure 2a). Pairwise *F_ST_* values using all loci revealed the most genetically divergent populations to be CRN (*F_ST_* range = 0.0021–0.0219), PPC (*F_ST_* range = 0.0014–0.0187), and one outgroup GSC (*F_ST_* range = 0.0014–0.0219) (Table S3). The divergence of CRN, PPC, and GSC was also evident for neutral loci, although *F_ST_* estimates were slightly lower (Table S4). Dendrograms of pairwise *F_ST_* values clearly separate CRN and PPC from the rest of the Sulu Sea populations using all loci and neutral loci; all *p*‐values for pairwise comparisons were <0.001 except for CRN‐MSJ and CRN‐NEG for both marker sets (Figure 3a,b). Excluding CRN and PPC, further structure is detected among the 6 Sulu Sea sites at all loci (*F_ST_* = 0.0016, *p* = .002), with significant *F_ST_* between TWI‐ROX at all loci (Table S3), but no further structure at neutral loci (Table S4).

**FIGURE 4:**
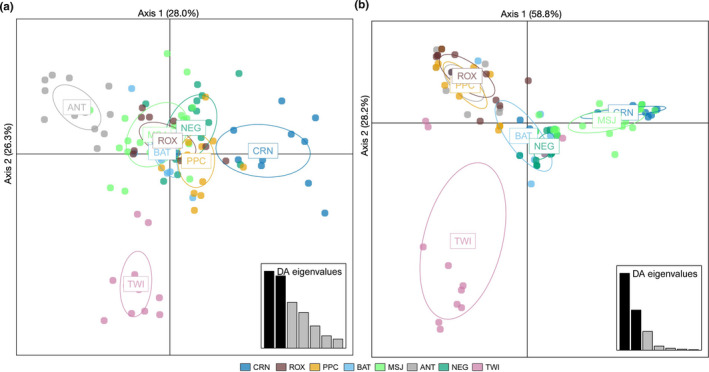
(a) Scatterplot of genetic clusters of Sulu Sea populations identified by DAPC based on neutral loci (1,643 SNPs) and sampling location information as a prior. Genetic clusters identified using outlier loci (12 SNPs) and sampling location information as a prior (b)

The DAPC using neutral loci shows separation of CRN, PPC, ANT, and TWI from the other Sulu Sea populations (Figure 4a). Based on 55 principal components, the first three discriminant functions explained 28.0%, 26.3%, and 16.7% of the variance, respectively. The first discriminant axis reveals the separation of CRN, PPC, and ANT, the second discriminant axis separates TWI, and the third axis (not shown) further separates PPC. While pairwise *F_ST_ p*‐values provide strong support for the separation of CRN and PPC, and to a lesser extent TWI, there is no evident support for ANT as a distinct population based on neutral loci. GENELAND recovered four genetic clusters for the neutral loci (Figure S1). Spatial patterns of clustering were broadly consistent with the results of *F_ST_* and DAPC analyses, with the recovery of CRN and PPC as distinct populations, but differed with the recovery of BAT as a distinct group. The rest of the sites were identified as a single cluster (MSJ, ANT, NEG, ROX, and TWI). An AMOVA testing a hypothesis of three genetic groups, (a) CRN, (b) PPC, and (c) the rest of the Sulu Sea populations, showed significant differentiation between groups (*F_CT_* = 0.017, *p* = .002; accounting for 1.7% of the total variance).

**FIGURE 5:**
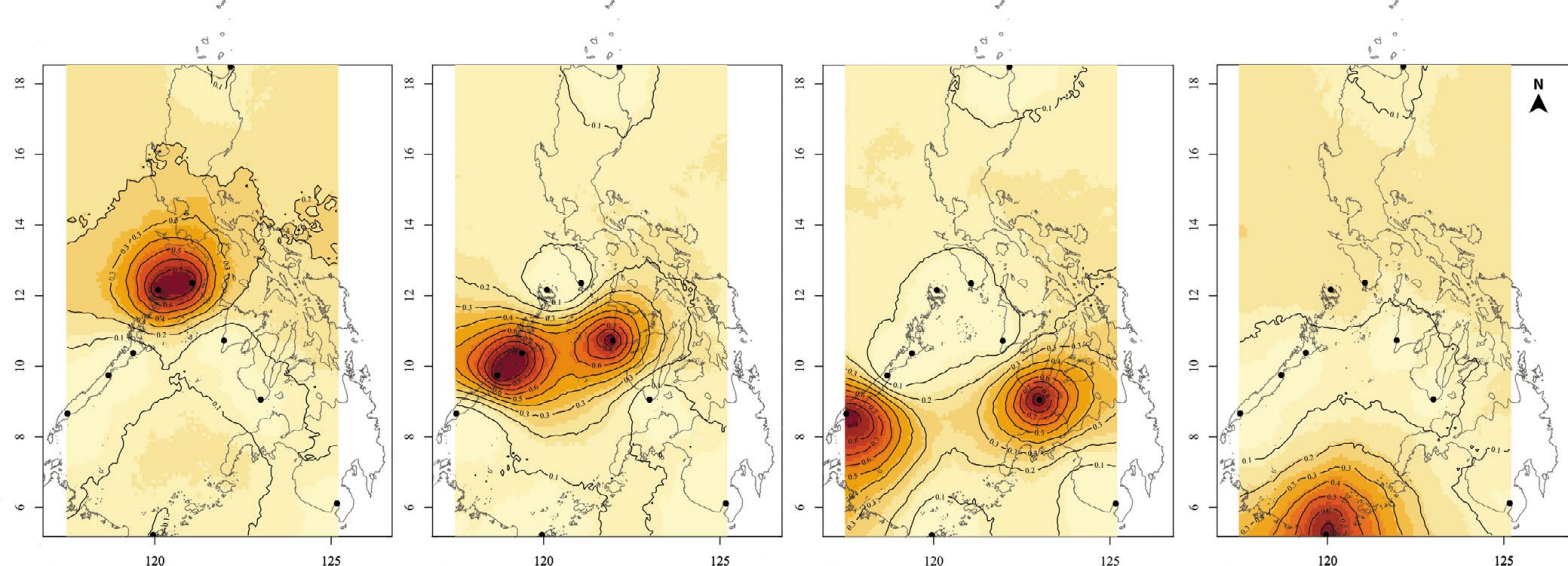
Genetic clusters of *Scylla olivacea* in the Sulu Sea based on GENELAND analysis of outlier loci. Posterior probability isoclines illustrate putative genetic landscapes for the Sulu Sea domain, where sites are represented by black dots, and darker colors indicate higher probabilities of membership to each of the six clusters identified across all sampling sites. Isoclines for the outgroup populations representing two genetic clusters are not shown

Outlier loci revealed pronounced genetic differentiation across the Sulu Sea (12 SNPs; *F_ST_* = 0.2390, *p* = .001). A dendrogram based on pairwise *F_ST_* estimates suggests four genetic clusters in the Sulu Sea: (a) CRN‐MSJ; (b) ROX‐PPC‐ANT; (c) BAT‐TWI; and (d) NEG (Figure 3c). Pairwise *F_ST_* among populations within each of the 3 clusters is not significant (FDR *p* > .05), that is, for ROX‐PPC‐ANT and BAT‐TWI, while between‐cluster comparisons are significant (FDR *p* < .05) (Table S5). The DAPC plot of 12 outlier loci (generated using 11 principal components following cross‐validation) suggests four genetic clusters exhibiting limited overlap in their 95% CI ellipses and recovered the same spatial structure as pairwise *F_ST_*, except that it clustered BAT and NEG, with TWI as a divergent population (Figure 4b). The first discriminant axis (58.8% of the total variance) establishes 3 groups: ROX‐PPC‐ANT‐TWI, BAT‐NEG, and MSJ‐CRN. The second discriminant axis (28.25% of total variance) separates TWI and establishes it as a fourth genetic group. GENELAND recovered four genetic clusters consistent with the DAPC grouping (Figure 5). AMOVA provides further support for the concordant groupings recovered by DAPC and GENELAND, with significant differentiation among the four groups (*F*_CT_ = 0.209; *p* = .001) accounting for 20.9% of the total observed variance. No further structure is detected among samples within groups (*F*_SC_ = 0.0168, *p* = .166). However, the discordance between pairwise *F_ST_* versus groupings recovered by DAPC and GENELAND for the southern sites BAT, NEG, and TWI populations may be influenced by missing data (19% over the outlier dataset). Thus, the clustering for these three populations should be approached with caution.

Considering the small number of outlier loci, missing data may have a big impact on the spatial genetic structure recovered by *F_ST_* and multivariate methods. To examine this further, we performed two separate analyses to handle missing data: (a) remove genotypes (individuals) with >25% missing data; and (b) impute missing data based on population frequencies as implemented in GenoDive v3.0 (Meirmans, 2020) (see Figure S2 for details). DAPC analysis of both datasets (genotypes removed and missing genotypes imputed) recovered the same four groups as the original dataset including missing data: CRN‐MSJ, ANT‐PPCR‐ROX, BAT‐NEG, and TWI (Figure S2). The consistent recovery of CRN‐MSJ (at 12°N) and ANT‐PPC‐ROX (at 9°N–11°N), as genetically distinct groups from BAT‐NEG and TWI (at 5°N to 8°N) by pairwise *F_ST_*, DAPC and GENELAND, provides support for a pattern of latitudinal structure of Sulu Sea populations based on outlier loci.

### 3.4 Gene flow estimates

Coalescent simulations using Migrate‐n revealed that the model representing bidirectional gene flow between East and West populations had the strongest support (Table 3). The immigration rate (*M*) of individuals from East into West was slightly higher than immigration of individuals from West into East, at 950.1 (95% highest posterior density (HPD): 942, 980.7) and 800.3 (95% HPD: 606–922), respectively. The model of unidirectional gene flow from East to West had a greater probability than unidirectional gene flow from West to East, while the model of panmixia had the lowest probability.

**TABLE 3:** Log Bayes factors (LBFs) calculated from the Bezier approximation scores for four models of migration between East and West Sulu Sea populations of *Scylla olivacea*

Model	Log (ml)	LBF	Rank
Full migration (bidirectional)	−2,266.35	0	1
East to West	−2,507.21	−340.86	2
West to East	−1,729.21	−562.86	3
Single genetic population (Panmixia)	−2,776.20	−609.85	4

Relative contemporary migration rates estimated from *divMigrate* indicate high levels of relative bidirectional gene flow (*m*) between eastern (MSJ‐ANT‐NEG) and western (ROX‐BAT) populations (*m* > 80%; Figure S3). Migration rates between TWI and the rest of the Sulu Sea sites ranged from 28% to 75% (mean = 43%), while migration rates involving the two divergent populations PPC and CRN and other populations were generally lower (*m* range = 32% to 69%, mean = 37%). No statistically significant asymmetries in gene flow patterns were detected (*n* = 1,000 bootstraps).

### 3.5 Genetic structure and environmental factors

Directional estimates of modeled larval dispersal generated seven asymmetric eigenvector maps (AEMs) representing predicted patterns of spatial genetic connectivity in the Sulu Sea. Using redundancy analysis (RDA), backward selection of the AEM variables identified two significant predictors (AEM6 and AEM7, with *p* > .05) (Table 4). Together, these two AEM eigenfunctions explained 13.3% of neutral genetic variation among sites (adjusted *R* ^2^; *p* = .035). The first RDA axis (RDA1) constituted the highest proportion of genetic variation in the response data (60.9%), which is only significant at the 10% level (*p* = .065), whereas RDA2 accounted for 39.1% of the total genetic variation (*p* = .292). Although AEM6 and AEM7 vectors were both selected to construct the model, individual testing of explanatory variables revealed that only AEM6 was significant (*p* < .001). The AEM6 eigenvector modeled Puerto Princesa (PPC) and Tawi‐Tawi (TWI) as separate units (Figure 2b).

**TABLE 4:** Summary of the redundancy analysis (RDA) of genetic data (neutral and outlier loci only) and environmental variables including the asymmetric eigenvector maps (AEMs), sea surface temperature (SST), and rainfall

	Significant variables	$R_{\text{adj}}^{2}$	*p*	RDA1	RDA2
AEM	*AEM6*	0.133	0.035	0.609*	0.391
	*AEM7*				
SST_mean_	*JUN*	0.763	0.041	**0.880**	0.102
	*AUG*				
	*SEP*				
	*DEC*				
SST_min_	*AUG*	0.692	0.050	**0.879**	0.103
	*SEP*				
	*OCT*				
	*DEC*				
SST_max_	*JUL*	0.738	0.048	0.884*	0.098
	*AUG*				
	*NOV*				
	*DEC*				
Rainfall	*MAR*	0.656	0.089	0.870	0.104
	*JUN*				
	*SEP*				
	*NOV*				

Note

Only those variables retained by backward selection and were significant (*p* < .05) are included in the final model. $R_{\text{adj}}^{2}$ represents the adjusted coefficient of determination with *p*‐values calculated using the analysis of variance (ANOVA; permutations = 999). The proportion of constrained RDA axes were presented in columns, and values in bold and with asterisk (*) indicate significant axes at 5% and 10% level, respectively.

Geographic distance shows no relationship with genetic variation for putatively neutral loci across all eight Sulu sites as revealed by distance‐based Moran's eigenvector map analysis (dbMEM; Dray et al., 2006). The procedure generated 7 nonsignificant dbMEM eigenvectors (*p* > .05), suggesting neutral genetic structure is not influenced by geographic distances. This was supported by additional Mantel tests resulting in a nonsignificant correlation between geographic distance and genetic distance (Mantel *r* = −0.086, *p* = .667). Examining eastern and western boundary populations separately, an emergent pattern of genetic distance increasing with geographic distance is observed for the eastern boundary populations (ANT, MSJ, NEG, TWI) although the relationship is not significant (Mantel *r* = 0.894, *p* = .083). Using the putatively neutral loci dataset, genetically divergent populations CRN, PPC, and GSC were estimated to have small effective population size (*N*_e_: CRN = 24.6–26.8, PPC = 10.7–11.2, GSC = 9.6–10.1) compared with other localities where *N*_e_ values range from 139.2 to very large (infinite) at 95% CI (Table S6).

Environmental data on SST and rainfall exhibited different levels of contribution to genetic differentiation and potential latitudinal adaptation of *S. olivacea* in the Sulu Sea. For SST, broadly concordant results were obtained from analyses of three explanatory SST variabilities (SST_mean_, SST_min_, and SST_max_), with each analysis having four independent vectors identified consisting of months mostly during the wet season (June through November, and December; Table 4). These explanatory variables contribute to 69.2%–76.3% of the total genetic variation among sites ($R_{\text{adj}}^{2}$ = 0.692–0.763, *p* < .05). RDA1 explained the highest fraction of genetic variation comprising 87.9%–88.4%, whereas RDA2 accounted for 9.8%–10.3%. Only SST_mean_ (*p* = .048) and SST_min_ (*p* = .049) showed statistically significant RDA1 axes, while SST_max_ is significant at 10% level (*p* = .056). Rainfall data explained a lower proportion of the variation ($R_{\text{adj}}^{2}$ = 0.656) despite having a similar number of significant variables contributing to genetic variation. The RDA model constructed using the rainfall vectors did not reveal significant correlation with genetic data (*p* = .089), which suggests that SST (Figure S4 showing SST_mean_ only) is a stronger predictor of the observed latitudinal genetic variation than rainfall.

## 4 DISCUSSION

This study employed a seascape genomics approach to examine environmental factors influencing genetic structure of *Scylla olivacea* populations in the Sulu Sea. Analysis of neutral markers revealed weak yet significant genetic differentiation. Moreover, genetic structure estimated from SNP markers is significantly correlated with genetic structure predicted from particle dispersal simulations, indicating the influence of ocean currents on gene flow. Geographic distance was not a significant predictor of genetic structure. Outlier loci revealed a pattern of latitudinal genetic structure suggesting local adaptation to latitudinal environmental gradients, with SST as a stronger predictor of adaptive divergence than rainfall. These results reveal basin‐scale genetic differentiation of *S. olivacea* populations in the Sulu Sea and insights on potential environmental drivers, information that is expected to be useful to support spatially explicit management and conservation interventions.

### 4.1 Genetic structure and connectivity in the Sulu Sea

The expectation of broad larval dispersal of *S. olivacea* based on life history features such as offshore spawning migration and a pelagic larval duration of 20–30 days (Ali et al., 2020) is consistent with larval dispersal simulations, which model that larvae have the potential to disperse widely across the Sulu Sea, a domain spanning 800 km north to south and 600 km maximum east to west. Surface circulation features modify the directionality of dispersal, which simulations show to be greater southward and eastward across the domain. The recovery of weak yet significant genetic differentiation among *S. olivacea* populations across the Sulu Sea demonstrates that dispersive life history features may not necessarily lead to widespread connectivity and genetic homogeneity. This study demonstrates the greater resolution afforded by SNP loci generated from RAD‐sequencing approaches to detect genetic differences. Using a panel of 1,655 SNPs and a reduced set of 1,643 putatively neutral SNPs, genetic structure was detected over a relatively smaller geographic area compared with a previous study based on mitochondrial DNA sequences reporting panmixia of *S. olivacea* from geographically disjunct sites along the western and eastern coasts of peninsular Malaysia (Strait of Malacca and South China Sea, respectively) (Rosly et al., 2013). While weak genetic differentiation was previously reported for *S. olivacea* based on microsatellite loci, the geographic coverage extended beyond the Sulu Sea, with a broader coverage across the Philippine archipelago (Paran & Ravago‐Gotanco, 2017).

This study adds to the growing body of literature reporting significant genetic differentiation for populations of marine organisms despite the potential for broad dispersal extending beyond the spatial scales covered by genetic sampling (Hauser & Carvalho, 2008). Weak yet significant genetic differentiation, with comparable estimates of low *F_ST_* values over sampling scales of hundreds of kilometers, has been reported for broadly dispersing species such as highly mobile fish (Atlantic cod, *F_ST_* = 0.004; Knutsen et al., 2003), or invertebrates with extensive larval duration periods such as red rock lobsters (*F_ST_* = 0.004; Iacchei et al., 2013) and spiny lobsters (*F_ST_* = 0.0016; Truelove et al., 2017). In the Adriatic Sea (800 km long, 200 km wide), a semi‐enclosed ocean basin comparable in area to the Sulu Sea (800 km long, 600 km wide), significant genetic differentiation was also reported for a range of organisms with similar bipartite life histories and broad dispersal potentials, such as the anchovy *Engraulis encrasicolus* (Bembo et al., 1996), shore crab *Carcinus aestuarii* (Schiavina et al., 2014), and peacock wrasse *Symphodus tinca* (Carreras et al., 2017), suggesting apparent barriers to dispersal even across distances of several hundred kilometers.

Coupling genetic analysis and oceanographic modeling approaches provide additional insights into genetic structure and connectivity of populations. For *S. olivacea,* geographic distance does not appear to be a factor in genetic structure across the Sulu Sea. Instead, oceanographic circulation appears to be a more significant driver of spatial patterns of dispersal and genetic structure. In particular, the predicted genetic pattern from AEM6, which identifies PPC and TWI as separate populations, is significantly correlated with the empirical allelic frequencies based on putatively neutral loci. This pattern is broadly consistent with the genetic analyses; that is, *F_ST_*‐based approaches reveal PPC as divergent from other Sulu Sea populations (Figures 2a, 3a,b). The separation of TWI, while not supported by pairwise *F_ST_ p*‐values after table‐wide FDR adjustment, is emergent in the DAPC plot (Figure 4a). The divergence of PPC and TWI may be due to self‐recruitment. TWI is modeled to have 100% self‐recruitment likely due to the southern Sulu gyre, which might promote entrainment, while self‐recruitment for PPC (49.7%) is relatively higher compared with the other Sulu Sea sites (22%–24%, except for BAT at 97.8%). Self‐recruitment estimates are not available for CRN, but we hypothesize high rates of self‐recruitment for this site considering its location in a deep embayment, which may preclude larval dispersal offshore.

Biophysical modeling reveals asymmetrical patterns of *S. olivacea* larval dispersal across the Sulu Sea, with greater dispersal from eastern to western boundary populations across the Sulu Sea. Genetic data, however, do not provide unequivocal support for this pattern, as approaches to infer long‐term and contemporary patterns and rates of migration both point to bidirectional gene flow as the most likely scenario. Nonetheless, greater westward dispersal may still be a plausible scenario. Coalescent analyses of gene flow reveal that while the bidirectional model has the greatest likelihood, migration rates are higher from East to West populations, and westward dispersal has greater support than a model of eastward dispersal. Moreover, given the high levels of gene flow between eastern and western populations revealed by similar allele frequency distributions exhibiting low genetic variance (*F_ST_* = 0), the hypothetical pool of migrants is expected to exhibit the same degree of genetic similarity to either West or East populations, and the accuracy of *divMigrate* estimates of directionality is likely to be low. Allele frequency‐based methods, particularly in moderate to high gene flow systems, fare poorly at distinguishing demographically significant connections and asymmetries (Waples, 1998). Simulated data show that under high gene flow scenarios corresponding to *F_ST_* ≥ 0.005 (which are still greater than the *F_ST_* estimates for *S. olivacea* across the Sulu Sea), the accuracy of directionality estimates does not exceed 50% (Sundqvist et al., 2016). Thus, taking into account the oceanographic data, combined with the challenge of estimating directionality against a background of high gene flow, asymmetrical dispersal from east to west cannot be discounted. Genetic studies for other species with similar limited adult movement, but shorter pelagic larval durations than *S. olivacea*, indicate limited gene flow between eastern and western boundary populations across the Sulu Sea. Genetic structure for the seahorse *Hippocampus spinosissimus* (Lourie et al., 2005), damselfish *Dascyllus aruanus* (Raynal et al., 2014), and sea cucumber *Holothuria scabra* (Ravago‐Gotanco & Kim, 2019) attributed limited dispersal across the Sulu Sea to a combination of oceanographic circulation features such as the Sulu Sea throughflow, the geographic distance across the Sulu Sea, and the absence of stepping‐stone reef habitats across the basin, as barriers to dispersal between eastern and western boundary populations. In contrast, larval dispersal simulations for three model organisms with varied dispersal potentials, a broadcast‐spawning coral *Acropora millepora*, sea urchin *Tripneustes gratilla*, and a reef fish *Epinephelus* sp., recovered three clusters in the Sulu Sea domain (North, Central, and Southern), but did not appear to indicate restricted dispersal between eastern and western boundary populations (Pata & Yniguez, 2019).

Population allele frequencies, while largely influenced by gene flow, may also reflect demographic changes (Whitlock & McCauley, 1999). The two most divergent populations, CRN and PPC, are characterized by low estimates of effective population size (CRN *N*_e_ = 24.6–26.8, PPC *N*_e_ = 10.7–11.2; Table S6), indicating the possible influence of genetic drift on allele frequency of small populations, which may lead to neutral divergence (Hare et al., 2011; Waples, 2010). Genetic divergence associated with low effective population sizes have been previously reported for other marine taxa, for example, red cusk‐eel *Genypterus chilensis* due to high fishing pressure (Córdova‐Alarcón et al., 2019), and population bottlenecks for *Gadus* morhua (Andreev et al., 2015) and *Epinephelus marginatus* (Buchholz‐Sørensen & Vella, 2016). The possible causes of low effective population sizes in CRN and PPC are not known. While high exploitation rates or diminished suitable habitat area may be underlying reasons for low population sizes, additional information from fishery data and habitat surveys (e.g., mangrove cover) is needed for a conclusive determination.

### 4.2 Latitudinal patterns of local adaptation of *Scylla olivacea* in the Sulu Sea

Environmental conditions can be agents of selection shaping the genotypic composition of local populations, with environmental heterogeneity resulting in increased adaptive potential, that is, an increased average fitness of organisms in their local environment than elsewhere (Hoban et al., 2016; Sanford & Kelly, 2011). As expected, *Scylla olivacea* populations exhibit pronounced genetic differentiation at outlier loci, with spatial patterns revealing latitudinal genetic structure across the Sulu Sea. Four genetic clusters were identified using multiple genetic approaches (*F_ST_*, DAPC, GENELAND), with AMOVA indicating significant differentiation among groups: (a) CRN‐MSJ, (b) ROX‐PPC‐ANT, (c) BAT‐NEG, and (d) TWI. SST variabilities can potentially explain the observed latitudinal genetic structure of *S. olivacea* in the Sulu Sea. The most significant variables (months) included in the RDA model cover the wet season (June–November) where the latitudinal thermal cline was the steepest. This significant association between latitudinal genetic structure and environmental variation during the wet season coincides with the reported peak spawning season of *Scylla* species in the Philippines (Arriola, 1940; Lebata et al., 2007), suggesting a biological response to environmental clines. Fine‐scale genetic structure recovered by adaptive polymorphisms likely reflects the influence of temporally variable latitudinal variations in environmental variables on *S. olivacea* during their early life stages, despite the potential for widespread dispersal and connectivity across the Sulu Sea.

The significant association of adaptive genetic divergence with SST variability in *S. olivacea* reflects the influence of temperature on life history characteristics of mud crabs. Water temperature and salinity are known key factors influencing larval development, growth, and survival of mud crabs (Baylon, 2011; Hill, 1974; Nurdiani & Zeng, 2007). Variability in temperature and salinity has been reported to influence reproductive characteristics. For instance, size at maturity in mud crabs was reported to vary with latitude, with smaller size at maturity in tropical regions hypothesized to be due to faster maturation in warmer waters (Alberts‐Hubatsch et al., 2015; Quinn & Kojis, 1987; Robertson & Kruger, 1994). Similar patterns of latitudinal variation in female size at maturity and fecundity were also reported for a closely related taxa, the burrowing mud crab, *Helise crasa* (Grapsidae) (Jones & Simons, 1981). *Scylla olivacea,* in particular, is known to exhibit latitudinal variation in the seasonality of peak spawning, reported to occur from July to November at latitudes between 9°N and 11°N (Koolkalya et al., 2006; Viswanathan et al., 2019), and March to September at higher latitudes (Ali et al., 2020; Ogawa et al., 2012). Latitudinal variability in temperature and salinity, through its influence on reproduction, larval survival, and development, is thus expected to play a significant role in the dynamics, genetic structure, and persistence of populations. Patterns of genetic differentiation associated with latitudinal gradients of temperature and salinity have been reported for several marine organisms across varying spatial scales. For instance, two major latitudinal clades were recovered in the North Atlantic snail *Nucella lapillus* along midcoastal Maine (between 43°N and 44°N; with water and air difference reaching up to 5–10°C), in which some of the genes involved in the genetic structure were associated with heat stress tolerance (Chu et al., 2014). A pattern of population structure was also found for a high gene flow marine fish *Larimichthys polyactis* in the Northwest Pacific marginal seas by using an outlier locus (e.g., heat‐shock protein), which is linked to local adaptation relating to seasonal variability in temperature between two regions separated by 1–2°C thermal difference between sites (Wang et al., 2013). For a marine diatom *Skeletonema marinoi*, a genetic break was found between the low‐salinity Baltic Sea and high‐salinity North Baltic Sea populations, despite the potential for migration between metapopulations based on oceanographic connectivity (Sjöqvist et al., 2015).

This study reports a pattern of latitudinal adaptive divergence associated with SST for populations of *S. olivacea* across the Sulu Sea. While we recovered a small number of putative outlier loci (12 SNP loci), these were consistently identified by two outlier loci detection methods. However, as these SNPs do not map to known functional genome regions, there is no basis to generate hypotheses regarding specific genes or gene regions potentially under adaptive selection. Further studies utilizing a larger number of SNP loci than what we were able to generate for this study (*n* = 1,665 loci in total) should be able to recover a proportionally greater number of putative outlier loci. Moreover, while the redundancy analysis indicates a significant association between SST and genetic variation based on outlier loci, analysis of an expanded set of environmental variables and genetic data in a more explicit genotype–environment association analysis is recommended for further studies. These are expected to contribute to greater insight into local adaptation and underlying factors influencing genetic differentiation, which is essential to understand the dynamics of populations particularly in the context of environmental variability.

### 4.3 Implications for management

Understanding spatial patterns of connectivity and the environmental drivers of local adaptation of populations represents key considerations for the design of effective, resilience‐based management interventions for fishery resources. For *S. olivacea*, basin‐scale genetic differentiation was detected at both the putatively neutral and outlier loci, reflecting the influence of evolutionary (e.g., genetic drift) and environmental processes (e.g., ocean currents, temperature, and salinity) on genotypic composition of populations in the Sulu Sea. The assessment of genetic diversity and connectivity of marine populations inferred from both neutral and outlier loci provides more holistic genetic information for fisheries management of populations (Carreras et al., 2017; Gagnaire et al., 2015; Nayfa & Zenger, 2016; Sandoval‐Castillo et al., 2018; Van Wyngaarden et al., 2017). In this study, we provide genetic resources (neutral and adaptive) to support the development of policy recommendations for management and conservation of *S. olivacea*.

From the perspective of neutral loci, *S. olivacea* populations in the Sulu Sea can be considered as a well‐connected metapopulation, with two divergent populations (Coron and Puerto Princesa) likely influenced by some restrictions to gene flow, but also genetic drift as a consequence of small effective population sizes. Populations with low *N*_e_ are particularly vulnerable to continued loss of genetic diversity and may need to be prioritized in restoration and conservation plans such as stock enhancement programs aimed at increasing yields beyond levels supported by natural recruitment (Bell et al., 2005). Stock enhancement programs initiated for the depleted mud crab *S. paramamosain* fishery in Japan report promising results toward increasing catch and population sizes after more than a decade of restoration efforts (Obata et al., 2006). Thus, this study recommends the development of management and conservation plans for vulnerable populations of *S. olivacea*, in Coron and Puerto Princesa, which are potentially facing higher rates of local extinction due to small effective population size.

Management strategies employing translocation of individuals should also be conducted with caution, with the view to maintain localized adaptive divergence among populations. In this context, evaluation of genetic variation using outlier markers is important, to detect signatures of local adaptation. In *S. olivacea*, we detected a pattern of genetic structure associated with environmental gradients such as sea surface temperature. These findings are important to consider in aquaculture practices and resource management interventions that rely on translocation of individuals across geographic locations. Successful adaptation is predicted to produce genotype–phenotype–environment associations, and translocation of locally adapted individuals may result in genetic environment mismatch and have significant impacts on fitness traits particularly beyond the limits of phenotypic plasticity (e.g., Kvingedal et al., 2010; Nayfa & Zenger, 2016). Thus, genetic information from this study can be used to identify sources of broodstock, which are potentially adapted to similar local environments. For example, individuals to be used in restocking the Coron population may be sourced from Mindoro, a nearby locality that is genetically similar to Coron. Likewise, the Puerto Princesa population may be restocked using individuals from an adjacent population in Roxas, Palawan, or a population across the basin, in Antique. This process may reduce outbreeding of genetically mismatched individuals that are locally adapted to different environmental conditions, which also limits the adverse effects on fitness and survival of these populations (Edmands, 2007; Edmands & Timmerman, 2003; Gharrett et al., 1999).

Overall, the results of this study can contribute to improve existing management and conservation plans for *S. olivacea* in the Philippines. *Scylla olivacea* was among the species included in a recent fisheries ordinance establishing guidelines limiting catch, trade, and transport of crablets, juvenile, and gravid individuals across the Philippines (Fisheries Administrative Order (FAO) 264s 2020; (BFAR, 2020). While not a priority species for aquaculture because of its aggressive behavior and smaller size than *S. serrata*, *S. olivacea* is the more abundant species and represents an important fishery resource that should be maintained and protected as a source of livelihood for small‐scale fishers across the Philippine archipelago. It is essential to augment genetics‐based approaches with other assessments of the fishery resource, to provide further insight into spatial distributions, genetic boundaries, and local adaptation in a rapidly changing marine environment, which are critical toward the design of management and conservation strategies.

## CONFLICT OF INTEREST

None declared.

## AUTHOR CONTRIBUTIONS

**Michael John R. Mendiola:** Conceptualization (equal); data curation (lead); formal analysis (lead); investigation (lead); methodology (equal); visualization (lead); writing–original draft (equal). **Rachel Ravago‐Gotanco:** Conceptualization (equal); formal analysis (supporting); funding acquisition (lead); methodology (equal); project administration (lead); supervision (lead); writing–original draft (equal).

## ETHICAL APPROVAL

*Scylla olivacea* is a commercially harvested species and was not identified as a regulated species at the time of sampling. For Palawan, collection and local transport were covered under Palawan Council for Sustainable Development (PCSD) local transport permits and Wildlife Gratuitous Permit (GP) no. 2016‐23. Export of material for DNA sequencing was covered under the Bureau of Fisheries and Aquatic Resources GP no. 2018‐0005.

### OPEN RESEARCH BADGES

This article has been awarded <Open Materials, Open Data>Badges. All materials and data are publicly accessible via the Open Science Framework at https://doi.org/10.5061/dryad.3xsj3txdz.

## Supporting information

Supplementary Material

## Data Availability

Raw demultiplexed sequence libraries in fastq format were archived in NCBI SRA (BioProject Accession # PRJNA662443). The corresponding filtered datasets and R scripts for analyses have been deposited in Dryad Digital Repository: https://doi.org/10.5061/dryad.3xsj3txdz
