# Supplementary material for: Genetic differentiation and signatures of local adaptation revealed by RADseq for a highly dispersive mud crab Scylla olivacea (Herbst, 1796) in the Sulu Sea
Source: Ecol Evol. 2021 May 4;11(12):7951–69. doi: 10.1002/ece3.7625 (PMC8216953; doi:10.1002/ece3.7625)
Supplement: Supplementary file 1 — Supplementary Material [file ECE3-11-7951-s001.docx]

Appendix

**Genetic differentiation and signatures of local adaptation revealed by RADseq for a highly-dispersive mud crab *Scylla olivacea* (Herbst, 1796) in the Sulu Sea**

Michael John R. Mendiola*, Rachel Ravago-Gotanco

Contents

[Table S1. Connectivity matrix based on the proportion of simulated larvae that were released from each source site (*i*) that disperse and settle in each destination site (*j*) after biophysical transport for one year. Dash symbol (-) indicates no particles were released at the source location and no information for settlement. Shaded cells show the percentage of local retention. 3](#_Toc68974365)

[Table S2. BLASTN alignment summary for 8 of 12 outlier loci detected using Bayescan and Arlequin. 4](#_Toc68974366)

[Table S3. Pairwise *F*_ST_ values of *S. olivacea* Sulu Sea and outgroup populations using all markers (1655 SNPs). Pairwise *F*_ST_ values (below diagonal) and *p*-values from tests of differentiation (above diagonal). Significant differences are highlighted in bold text (*p* ≤ 0.05). All *p*-values were calculated using 10,000 bootstraps, adjusted for false discovery rate (FDR). 7](#_Toc68974367)

[Table S4. Pairwise *F*_ST_ values of *S. olivacea* Sulu Sea and outgroup populations using neutral markers (1643 SNPs). Pairwise *F*_ST_ values (below diagonal) and *p*-values from tests of differentiation (above diagonal). Significant differences are highlighted in bold text (*p* ≤ 0.05). All *p*-values were calculated using 10,000 bootstraps, adjusted for false discovery rate (FDR). 8](#_Toc68974368)

[Table S5. Pairwise *F*_ST_ values of *S. olivacea* Sulu Sea and outgroup populations using outlier markers (12 SNPs). Pairwise *F*_ST_ values (below diagonal) and *p*-values from tests of differentiation (above diagonal). Significant differences are highlighted in bold text (*p* ≤ 0.05). All *p*-values were calculated using 10,000 bootstraps, adjusted for false discovery rate (FDR). 9](#_Toc68974369)

[Table S6. Estimated contemporary effective population size (*N*_e_) of *S. olivacea* populations using the neutral SNP panel (1,643 loci). Contemporary *N*_e_ among local populations was calculated using NeEstimator v2.1. 10](#_Toc68974370)

[Figure S1. Genetic clusters of *S. olivacea* in the Sulu Sea based on GENELAND analysis of neutral loci (K = 4). Posterior probability isoclines illustrate putative genetic landscapes for the Sulu Sea domain, where sites are represented by black dots, and darker colors indicate higher probabilities of membership to each of the four genetic clusters identified across all sampling sites. Isoclines for the outgroup populations representing two genetic clusters are not shown. 11](#_Toc68974371)

[Figure S2. DAPC scatterplot of Sulu Sea populations based on additional analyses of outlier loci datasets with different treatments of missing data. (a) Missing data excluded by removal of genotypes (individuals) with missing data at more than 3 loci. Twenty-three individuals were excluded (n = 93 individuals retained), missing data reduced to 8.1% from 19.1%, 10 principal components retained after cross-validation. The two axes recovered 87.62% of the total variance (67.34% for axis 1, 20.28% for axis 2). (b) Missing data imputed based on population allele frequencies in GenoDive (Meirmans 2020), 9 principal components retained after cross-validation; the two axes recovered 89.08% of the total variance (65.60% for axis 1, 23.48% for axis 2). Both scatterplots recover four broadly concordant groupings of Sulu Sea populations: CRN-MSJ; ANT-PPC-ROX, BAT-NEG and TWI. 12](#_Toc68974372)

[Figure S3. Relative migration rates of 5 *S. olivacea* populations in the Sulu Sea calculated with divMigrate and based on G_ST_. 13](#_Toc68974373)

[Figure S4. Maps of average sea surface temperature (SST) in the Sulu Sea basin from January to December. Average SST was measured along 5-12**°**N and 116-124**°**E from 1987 to 2005. Data provided by the NOAA ESRL Physical Sciences Laboratory, Boulder, Colorado, USA, from their website at http://psl.noaa.gov/. 14](#_Toc68974374)

Table S1. Connectivity matrix based on the proportion of simulated larvae that were released from each source site (*i*) that disperse and settle in each destination site (*j*) after biophysical transport for one year. Dash symbol (-) indicates no particles were released at the source location and no information for settlement. Shaded cells show the percentage of local retention.

|  | **CRN** | **PPC** | **ROX** | **BAT** | **MSJ** | **ANT** | **NEG** | **TWI** |
| --- | --- | --- | --- | --- | --- | --- | --- | --- |
| **CRN** | *-* | *-* | *-* | *-* | *-* | *-* | *-* | *-* |
| **PPC** | *0.001* | *0.497* | *0.273* | *0.228* | *0.000* | *0.000* | *0.000* | *0.000* |
| **ROX** | *0.002* | *0.601* | *0.225* | *0.165* | *0.000* | *0.000* | *0.004* | *0.003* |
| **BAT** | *0.000* | *0.004* | *0.001* | *0.978* | *0.000* | *0.000* | *0.000* | *0.018* |
| **MSJ** | *0.000* | *0.377* | *0.117* | *0.050* | *0.238* | *0.127* | *0.092* | *0.000* |
| **ANT** | *0.000* | *0.454* | *0.081* | *0.078* | *0.000* | *0.255* | *0.104* | *0.027* |
| **NEG** | *0.000* | *0.176* | *0.005* | *0.262* | *0.000* | *0.000* | *0.247* | *0.311* |
| **TWI** | *0.000* | *0.000* | *0.000* | *0.000* | *0.000* | *0.000* | *0.000* | *1.000* |

## Table S2. BLASTN alignment summary for 8 of 12 outlier loci detected using Bayescan and Arlequin.

| **Locus  Name** | **Locus ID** | **RAD Tag/ Sequence** | **Accession** | **Description** | [**%  Identity**](https://blast.ncbi.nlm.nih.gov/Blast.cgi?CMD=Get&ADV_VIEW=yes&ADV_VIEW=on&ALIGNMENTS=100&ALIGNMENT_VIEW=Pairwise&DATABASE_SORT=0&DESCRIPTIONS=100&DYNAMIC_FORMAT=on&FIRST_QUERY_NUM=0&FORMAT_NUM_ORG=1&FORMAT_OBJECT=Alignment&FORMAT_PAGE_TARGET=&FORMAT_TYPE=HTML&GET_SEQUENCE=yes&I_THRESH=&LINE_LENGTH=60&MASK_CHAR=2&MASK_COLOR=1&NUM_OVERVIEW=100&PAGE=Nucleotides&QUERY_INDEX=0&QUERY_NUMBER=0&RESULTS_PAGE_TARGET=&RID=UD7VSX3G014&SHOW_LINKOUT=yes&SHOW_OVERVIEW=yes&STEP_NUMBER=&WORD_SIZE=11&ADV_VIEW=on&DISPLAY_SORT=3&HSP_SORT=3) | [**Max Score**](https://blast.ncbi.nlm.nih.gov/Blast.cgi?CMD=Get&ADV_VIEW=yes&ADV_VIEW=on&ALIGNMENTS=100&ALIGNMENT_VIEW=Pairwise&DATABASE_SORT=0&DESCRIPTIONS=100&DYNAMIC_FORMAT=on&FIRST_QUERY_NUM=0&FORMAT_NUM_ORG=1&FORMAT_OBJECT=Alignment&FORMAT_PAGE_TARGET=&FORMAT_TYPE=HTML&GET_SEQUENCE=yes&I_THRESH=&LINE_LENGTH=60&MASK_CHAR=2&MASK_COLOR=1&NUM_OVERVIEW=100&PAGE=Nucleotides&QUERY_INDEX=0&QUERY_NUMBER=0&RESULTS_PAGE_TARGET=&RID=UD7VSX3G014&SHOW_LINKOUT=yes&SHOW_OVERVIEW=yes&STEP_NUMBER=&WORD_SIZE=11&ADV_VIEW=on&DISPLAY_SORT=1&HSP_SORT=1) | [**Total Score**](https://blast.ncbi.nlm.nih.gov/Blast.cgi?CMD=Get&ADV_VIEW=yes&ADV_VIEW=on&ALIGNMENTS=100&ALIGNMENT_VIEW=Pairwise&DATABASE_SORT=0&DESCRIPTIONS=100&DYNAMIC_FORMAT=on&FIRST_QUERY_NUM=0&FORMAT_NUM_ORG=1&FORMAT_OBJECT=Alignment&FORMAT_PAGE_TARGET=&FORMAT_TYPE=HTML&GET_SEQUENCE=yes&I_THRESH=&LINE_LENGTH=60&MASK_CHAR=2&MASK_COLOR=1&NUM_OVERVIEW=100&PAGE=Nucleotides&QUERY_INDEX=0&QUERY_NUMBER=0&RESULTS_PAGE_TARGET=&RID=UD7VSX3G014&SHOW_LINKOUT=yes&SHOW_OVERVIEW=yes&STEP_NUMBER=&WORD_SIZE=11&ADV_VIEW=on&DISPLAY_SORT=2&HSP_SORT=1) | [**Query Cover**](https://blast.ncbi.nlm.nih.gov/Blast.cgi?CMD=Get&ADV_VIEW=yes&ADV_VIEW=on&ALIGNMENTS=100&ALIGNMENT_VIEW=Pairwise&DATABASE_SORT=0&DESCRIPTIONS=100&DYNAMIC_FORMAT=on&FIRST_QUERY_NUM=0&FORMAT_NUM_ORG=1&FORMAT_OBJECT=Alignment&FORMAT_PAGE_TARGET=&FORMAT_TYPE=HTML&GET_SEQUENCE=yes&I_THRESH=&LINE_LENGTH=60&MASK_CHAR=2&MASK_COLOR=1&NUM_OVERVIEW=100&PAGE=Nucleotides&QUERY_INDEX=0&QUERY_NUMBER=0&RESULTS_PAGE_TARGET=&RID=UD7VSX3G014&SHOW_LINKOUT=yes&SHOW_OVERVIEW=yes&STEP_NUMBER=&WORD_SIZE=11&ADV_VIEW=on&DISPLAY_SORT=4&HSP_SORT=0) | [**E value**](https://blast.ncbi.nlm.nih.gov/Blast.cgi?CMD=Get&ADV_VIEW=yes&ADV_VIEW=on&ALIGNMENTS=100&ALIGNMENT_VIEW=Pairwise&DATABASE_SORT=0&DESCRIPTIONS=100&DYNAMIC_FORMAT=on&FIRST_QUERY_NUM=0&FORMAT_NUM_ORG=1&FORMAT_OBJECT=Alignment&FORMAT_PAGE_TARGET=&FORMAT_TYPE=HTML&GET_SEQUENCE=yes&I_THRESH=&LINE_LENGTH=60&MASK_CHAR=2&MASK_COLOR=1&NUM_OVERVIEW=100&PAGE=Nucleotides&QUERY_INDEX=0&QUERY_NUMBER=0&RESULTS_PAGE_TARGET=&RID=UD7VSX3G014&SHOW_LINKOUT=yes&SHOW_OVERVIEW=yes&STEP_NUMBER=&WORD_SIZE=11&ADV_VIEW=on&DISPLAY_SORT=0&HSP_SORT=0) | **Length** |
| --- | --- | --- | --- | --- | --- | --- | --- | --- | --- | --- |
| SNP_489 | 4533 | AATTCTTACTCCATAAAAAGGTCAGAAGTCAGGGGTCAATACAGCAGTTCTGGTCAGGTTTCTCGTCCCCTCTCCCCTGTGCGTGGCGCGTGGGCTCATGAAAAAGAGACGGGTTACGTGCAAGGCATGGCAACACAAAGCACTCT | [LR697120.1](https://www.ncbi.nlm.nih.gov/nucleotide/LR697120.1?report=genbank&log$=nucltop&blast_rank=1&RID=UD7VSX3G014) | [*Chanos chanos* genome assembly, chromosome: 15](https://blast.ncbi.nlm.nih.gov/Blast.cgi#alnHdr_1721133936) | 96.15% | 43.7 | 87.3 | 17% | 3.7 | 20460689 |
| SNP_9112 | 75640 | AATTCCCACCGACAAGCCTGCATGGTGGCAGGGACTGGGCGATGTGTGAAGGTTCATTAAGGCAGAGATGAGGCTTTCATGCCCACTAGTGACCGTGGAGGCGCGAGTGATATTAGCAGAGGACCTGGCAGTGTCTGTGGTCGATT | [LR664371.1](https://www.ncbi.nlm.nih.gov/nucleotide/LR664371.1?report=genbank&log$=nucltop&blast_rank=1&RID=UD9SU755014) | [*Coregonus* sp.](https://blast.ncbi.nlm.nih.gov/Blast.cgi#alnHdr_1711366980)  ['balchen'](https://blast.ncbi.nlm.nih.gov/Blast.cgi#alnHdr_1711366980)  [genome](https://blast.ncbi.nlm.nih.gov/Blast.cgi#alnHdr_1711366980)  [assembly,](https://blast.ncbi.nlm.nih.gov/Blast.cgi#alnHdr_1711366980)  [chromosome:](https://blast.ncbi.nlm.nih.gov/Blast.cgi#alnHdr_1711366980)  [28](https://blast.ncbi.nlm.nih.gov/Blast.cgi#alnHdr_1711366980) | 80.85% | 45.5 | 45.5 | 32% | 1.1 | 46671285 |
| SNP_1711 | 12986 | AATTCTCTGAACCAAGTCCTGAGCCTGTGAAGTGTAAGAGACGCATCAACTTTGGCGTCGGATACGTTGTGTCGCCAGCGCCCGTGGCAGTGGCGCGCCGCAATGCACGAGAGAGGAACCGCGTGAAGCAAGTCAACAACGGGTTC | [XM_027372453.1](https://www.ncbi.nlm.nih.gov/nucleotide/XM_027372453.1?report=genbank&log$=nucltop&blast_rank=13&RID=UD8YS1VB015) | [PREDICTED: *Penaeus vannamei* achaete-scute complex protein T5-like (LOC113820168), mRNA](https://blast.ncbi.nlm.nih.gov/Blast.cgi#alnHdr_1536077955) | 85.14% | 167 | 167 | 99% | 1.00E-37 | 2250 |
| SNP_5137 | 41962 | AATTCTGAGCCAATGGTAGCCGCACTAGCCAGGCTTCCTATGCAAGACACAAGAGAAGAGAAAGGTGTTTCTCACGGAGCACGGAAGCGAGAGAGCGTAAGGCAGAATACCTCAAGAGAAACCTAGAATCATCAAGATAGTGTTTT | [LR697112.1](https://www.ncbi.nlm.nih.gov/nucleotide/LR697112.1?report=genbank&log$=nucltop&blast_rank=3&RID=UD9NTWYT014) | [*Chanos chanos* genome assembly, chromosome: 7](https://blast.ncbi.nlm.nih.gov/Blast.cgi#alnHdr_1721134131) | 86.49% | 42.8 | 85.5 | 23% | 3.7 | 49187548 |
| SNP_9743 | 81546 | AATTCAGAACTCATAATGATCGATCTTTGGGTAGTACTGAGACCACTCACACACCACACACCGCGATAGCGAGGCCACAATCCTTCGGGTTACATCCCGTACCTACTTACTGCTAGGTGAACACGGCCTGCATATTAAGAGGCTTG | [EU794021.1](https://www.ncbi.nlm.nih.gov/nucleotide/EU794021.1?report=genbank&log$=nucltop&blast_rank=6&RID=UD9XJ8S2014) | [*Portunus trituberculatus* clone CGT4A9 microsatellite sequence](https://blast.ncbi.nlm.nih.gov/Blast.cgi#alnHdr_193075686) | 87.76% | 120 | 120 | 66% | 2.00E-23 | 302 |
| SNP_2106 | 15636 | AATTCGTAAGGCTCCATTTTGTGTGTGTGTGTGTGTGTGTGTGTTTTCAAATCTCCCTTCAATAACCCAAGTCTTAACTCCCAACACTTGTCGAGTAACGTATGGCATCAGAAAATAAGGTGTACAAGGATGGCATTAGCAGGTGC | [LR584440.1](https://www.ncbi.nlm.nih.gov/nucleotide/LR584440.1?report=genbank&log$=nucltop&blast_rank=2&RID=UD99K35S015) | [*Salmo trutta* genome assembly, chromosome: 22](https://blast.ncbi.nlm.nih.gov/Blast.cgi#alnHdr_1614056738) | 97.30% | 63.5 | 63.5 | 25% | 4.00E-06 | 52209666 |
| SNP_2231 | 16555 | AATTCAGTGACACAAAAGGAGCACTTGCATGTATTCTTAAAAAAAACATATTTTAGAGGGAGAAAGGAGCACTTACATGTATTATTAAATGGATTAAGAACACTCAATAGGAAGAAGCAGGAAAGTATATAACAACAGGGAATGGG | [LR537132.1](https://www.ncbi.nlm.nih.gov/nucleotide/LR537132.1?report=genbank&log$=nucltop&blast_rank=9&RID=UD9D28KG015) | [*Sparus aurata* genome assembly, chromosome: 12](https://blast.ncbi.nlm.nih.gov/Blast.cgi#alnHdr_1711374622) | 100.00% | 42.8 | 42.8 | 15% | 3.7 | 30193437 |
| SNP_4648 | 38327 | AATTCCTTCCAGCTACAACACAGTATTGTTGTGAAGAGCACGAGAGGAGTGATAACTTCTAGCTTTACTCAAAGACAGCCCATTTCAGTATCGGTATACTGAAAACACAGCCGTGGCATCTAAGACAACACTGCGTTACCATCTCC | [CR855860.7](https://www.ncbi.nlm.nih.gov/nucleotide/CR855860.7?report=genbank&log$=nucltop&blast_rank=1&RID=UD9KN3J2014) | [Zebrafish DNA sequence from clone CH211-239D6 in linkage group 19, complete sequence](https://blast.ncbi.nlm.nih.gov/Blast.cgi#alnHdr_60302548) | 100.00% | 44.6 | 44.6 | 16% | 1.1 | 90948 |

## Table S3. Pairwise *F*_ST_ values of *S. olivacea* Sulu Sea and outgroup populations using all markers (1655 SNPs). Pairwise *F*_ST_ values (below diagonal) and *p*-values from tests of differentiation (above diagonal). Significant differences are highlighted in bold text (*p* ≤ 0.05). All *p*-values were calculated using 10,000 bootstraps, adjusted for false discovery rate (FDR).

| **Site** | **CRN** | **ROX** | **PPC** | **BAT** | **MSJ** | **ANT** | **NEG** | **TWI** | **CGY** | **GSC** |
| --- | --- | --- | --- | --- | --- | --- | --- | --- | --- | --- |
| **CRN** |  | *0.0000* | *0.0000* | *0.0000* | *0.1211* | *0.0000* | *0.1049* | *0.0000* | *0.0119* | *0.0000* |
| **ROX** | ***0.0107*** |  | *0.0000* | *0.6010* | *0.1079* | *0.5834* | *0.1025* | *0.0260* | *0.1079* | *0.0000* |
| **PPC** | ***0.0187*** | ***0.0110*** |  | *0.0056* | *0.0000* | *0.0000* | *0.0000* | *0.0000* | *0.0000* | *0.3304* |
| **BAT** | ***0.0100*** | *-0.0004* | ***0.0075*** |  | *0.5834* | *0.6177* | *0.2216* | *0.4130* | *0.1498* | *0.0007* |
| **MSJ** | *0.0021* | *0.0029* | ***0.0102*** | *-0.0001* |  | *0.6478* | *0.3304* | *0.0817* | *0.1141* | *0.0000* |
| **ANT** | ***0.0105*** | *-0.0001* | ***0.0103*** | *-0.0005* | *-0.0006* |  | *0.6203* | *0.0571* | *0.1049* | *0.0000* |
| **NEG** | *0.0047* | *0.0036* | ***0.0107*** | *0.0024* | *0.0010* | *-0.0005* |  | *0.0723* | *0.1049* | *0.0000* |
| **TWI** | ***0.0107*** | ***0.0055*** | ***0.0144*** | *0.0011* | *0.0029* | *0.0035* | *0.0037* |  | *0.0119* | *0.0000* |
| **CGY** | ***0.0071*** | *0.0032* | ***0.0082*** | *0.0030* | *0.0023* | *0.0028* | *0.0031* | *0.0054* |  | *0.0000* |
| **GSC** | ***0.0219*** | ***0.0145*** | *0.0014* | ***0.0103*** | ***0.0105*** | ***0.0167*** | ***0.0151*** | ***0.0192*** | ***0.0092*** |  |

## Table S4. Pairwise *F*_ST_ values of *S. olivacea* Sulu Sea and outgroup populations using neutral markers (1643 SNPs). Pairwise *F*_ST_ values (below diagonal) and *p*-values from tests of differentiation (above diagonal). Significant differences are highlighted in bold text (*p* ≤ 0.05). All *p*-values were calculated using 10,000 bootstraps, adjusted for false discovery rate (FDR).

| **Site** | **CRN** | **ROX** | **PPC** | **BAT** | **MSJ** | **ANT** | **NEG** | **TWI** | **CGY** | **GSC** |
| --- | --- | --- | --- | --- | --- | --- | --- | --- | --- | --- |
| **CRN** |  | *0.0003* | *0.0000* | *0.0009* | *0.1708* | *0.0000* | *0.1474* | *0.0000* | *0.0327* | *0.0000* |
| **ROX** | ***0.0078*** |  | *0.0000* | *0.7816* | *0.4799* | *0.6195* | *0.3292* | *0.1344* | *0.2694* | *0.0000* |
| **PPC** | ***0.0160*** | ***0.0110*** |  | *0.0182* | *0.0000* | *0.0000* | *0.0000* | *0.0000* | *0.0003* | *0.7296* |
| **BAT** | ***0.0081*** | *-0.0016* | ***0.0063*** |  | *0.7792* | *0.8419* | *0.2694* | *0.4799* | *0.2726* | *0.0008* |
| **MSJ** | *0.0021* | *0.0005* | ***0.0080*** | *-0.0012* |  | *0.9823* | *0.5729* | *0.2694* | *0.4799* | *0.0000* |
| **ANT** | ***0.0075*** | *-0.0001* | ***0.0104*** | *-0.0019* | *-0.0031* |  | *0.9458* | *0.2587* | *0.2488* | *0.0000* |
| **NEG** | *0.0029* | *0.0015* | ***0.0089*** | *0.0023* | *0.0001* | *-0.0025* |  | *0.1158* | *0.2488* | *0.0000* |
| **TWI** | ***0.0085*** | *0.0037* | ***0.0128*** | *0.0009* | *0.0015* | *0.0018* | *0.0034* |  | *0.0327* | *0.0000* |
| **CGY** | ***0.0044*** | *0.0019* | ***0.0073*** | *0.0021* | *0.0004* | *0.0019* | *0.0021* | *0.0046* |  | *0.0000* |
| **GSC** | ***0.0207*** | ***0.0122*** | *-0.0008* | ***0.0091*** | ***0.0096*** | ***0.0145*** | ***0.0140*** | ***0.0186*** | ***0.0076*** |  |

## Table S5. Pairwise *F*_ST_ values of *S. olivacea* Sulu Sea and outgroup populations using outlier markers (12 SNPs). Pairwise *F*_ST_ values (below diagonal) and *p*-values from tests of differentiation (above diagonal). Significant differences are highlighted in bold text (*p* ≤ 0.05). All *p*-values were calculated using 10,000 bootstraps, adjusted for false discovery rate (FDR).

| **Site** | **CRN** | **ROX** | **PPC** | **BAT** | **MSJ** | **ANT** | **NEG** | **TWI** | **CGY** | **GSC** |
| --- | --- | --- | --- | --- | --- | --- | --- | --- | --- | --- |
| **CRN** |  | *0.0097* | *0.0000* | *0.0000* | *0.1388* | *0.0000* | *0.0000* | *0.0000* | *0.0000* | *0.0028* |
| **ROX** | ***0.3668*** |  | *0.6094* | *0.0000* | *0.0000* | *0.4717* | *0.0000* | *0.0000* | *0.0097* | *0.0000* |
| **PPC** | ***0.3714*** | *-0.0019* |  | *0.0002* | *0.0000* | *0.8255* | *0.0000* | *0.0000* | *0.0008* | *0.0002* |
| **BAT** | ***0.3071*** | ***0.2027*** | ***0.2055*** |  | *0.0000* | *0.0000* | *0.0000* | *0.3524* | *0.0000* | *0.0012* |
| **MSJ** | *0.0113* | ***0.3150*** | ***0.3160*** | ***0.1906*** |  | *0.0000* | *0.0000* | *0.0000* | *0.0000* | *0.0000* |
| **ANT** | ***0.3791*** | *0.0024* | *-0.0066* | ***0.2155*** | ***0.3268*** |  | *0.0008* | *0.0000* | *0.0000* | *0.0008* |
| **NEG** | ***0.3762*** | ***0.3662*** | ***0.3500*** | ***0.0642*** | ***0.2099*** | ***0.3614*** |  | *0.0426* | *0.0088* | *0.0315* |
| **TWI** | ***0.3303*** | ***0.2615*** | ***0.2524*** | *0.0444* | ***0.2271*** | ***0.2579*** | ***0.1068*** |  | *0.0000* | *0.0187* |
| **CGY** | ***0.3771*** | ***0.1934*** | ***0.1598*** | ***0.1758*** | ***0.2921*** | ***0.1438*** | ***0.2535*** | ***0.1665*** |  | *0.0011* |
| **GSC** | ***0.2140*** | ***0.2962*** | ***0.2877*** | ***0.2136*** | ***0.1590*** | ***0.2964*** | ***0.2661*** | ***0.1296*** | ***0.2527*** |  |

## Table S6. Estimated contemporary effective population size (*N*_e_) of *S. olivacea* populations using the neutral SNP panel (1,643 loci). Contemporary *N*_e_ among local populations was calculated using NeEstimator v2.1.

| Population Name  (N = Number of individuals) | **CRN**  (N =14) | **ROX**  (N = 13) | **PPC**  (N = 15) | **BAT**  (N = 12) | **MSJ**  (N = 22) | **ANT**  (N = 13) | **NEG**  (N = 15) | **TWI**  (N = 12) | **CGY**  (N = 15) | **GSC**  (N = 15) |
| --- | --- | --- | --- | --- | --- | --- | --- | --- | --- | --- |
| Estimated N_e_ at Pcrit 0.05 | 25.7 | ∞ | 10.9 | ∞ | 154.7 | ∞ | ∞ | ∞ | 368.0 | 9.8 |
| 95% CIs for N_e_ (Parametric) | 24.6 – 26.8 | ∞ | 10.7 – 11.2 | ∞ | 139.2 – 173.8 | 1015.7 – ∞ | 3426.7 – ∞ | ∞ | 252.2 – 673.5 | 9.6 –  10.1 |
| Estimated N_e_ at Pcrit 0.01 | 33.7 | ∞ | 15.7 | ∞ | 219.9 | ∞ | ∞ | ∞ | 1050.9 | 14.1 |
| 95% CIs for N_e_ (Parametric) | 32.3 – 35.3 | ∞ | 15.3 – 16.1 | ∞ | 193.4 – 254.7 | ∞ | ∞ | ∞ | 500.9 –  ∞ | 13.8 – 14.5 |


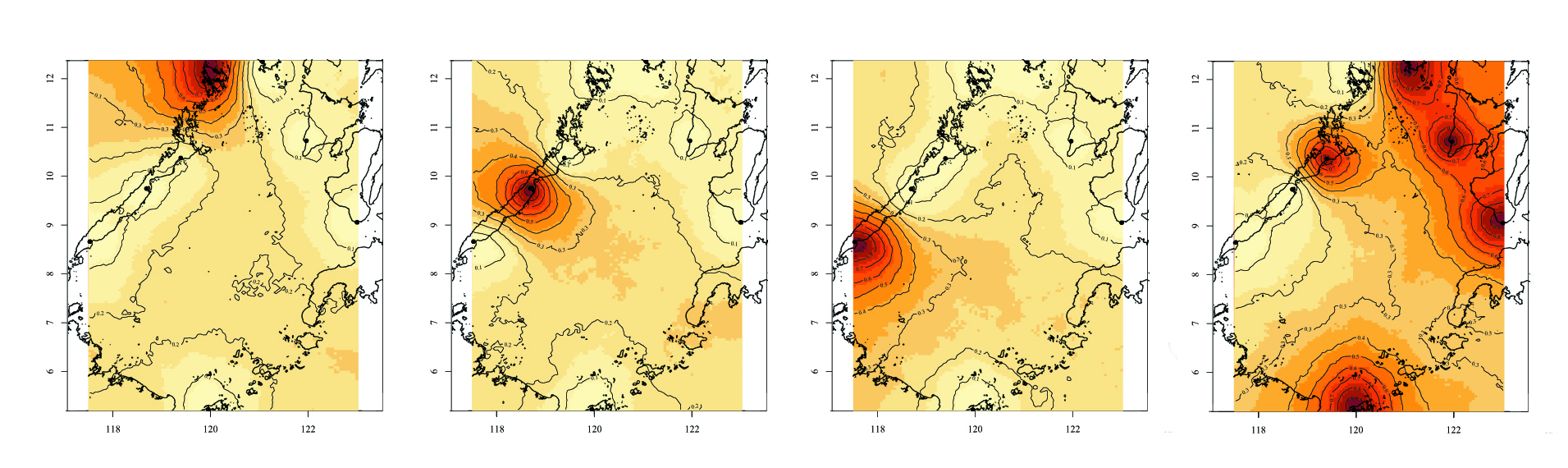


## Figure S1. Genetic clusters of *S. olivacea* in the Sulu Sea based on GENELAND analysis of neutral loci (K = 4). Posterior probability isoclines illustrate putative genetic landscapes for the Sulu Sea domain, where sites are represented by black dots, and darker colors indicate higher probabilities of membership to each of the four genetic clusters identified across all sampling sites. Isoclines for the outgroup populations representing two genetic clusters are not shown.

(a) (b)


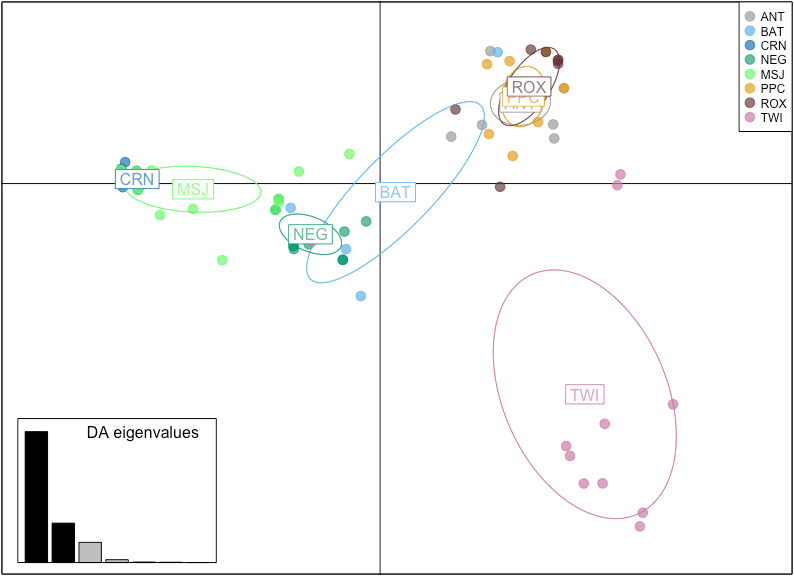

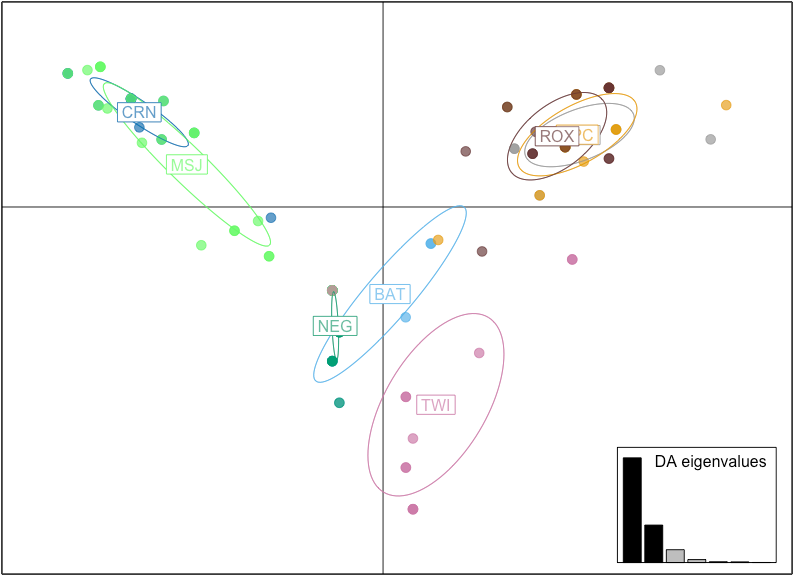


## Figure S2. DAPC scatterplot of Sulu Sea populations based on additional analyses of outlier loci datasets with different treatments of missing data. (a) Missing data excluded by removal of genotypes (individuals) with missing data at more than 3 loci. Twenty-three individuals were excluded (n = 93 individuals retained), missing data reduced to 8.1% from 19.1%, 10 principal components retained after cross-validation. The two axes recovered 87.62% of the total variance (67.34% for axis 1, 20.28% for axis 2). (b) Missing data imputed based on population allele frequencies in GenoDive (Meirmans 2020), 9 principal components retained after cross-validation; the two axes recovered 89.08% of the total variance (65.60% for axis 1, 23.48% for axis 2). Both scatterplots recover four broadly concordant groupings of Sulu Sea populations: CRN-MSJ; ANT-PPC-ROX, BAT-NEG and TWI.


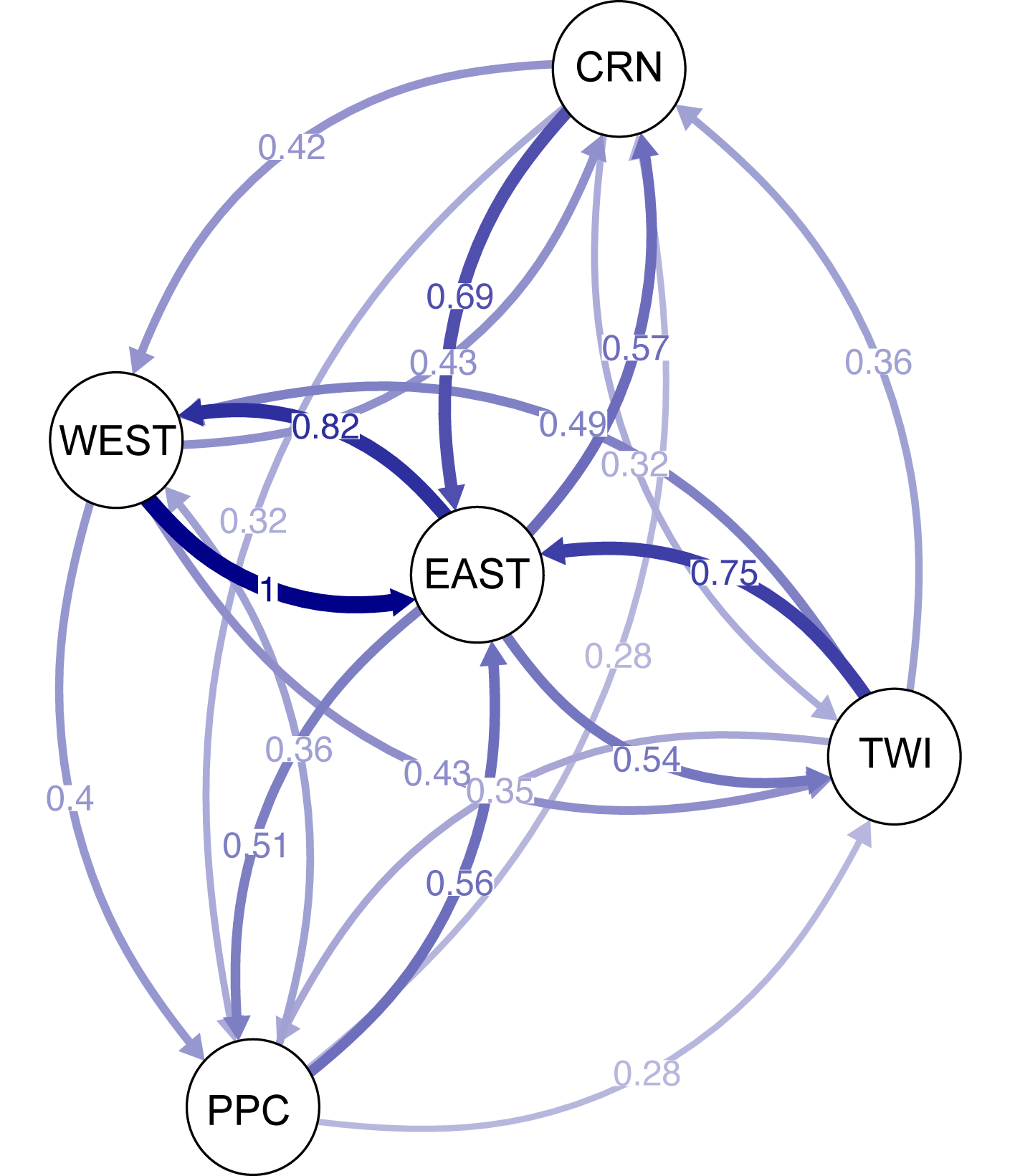


## [Figure S3. Relative migration rates of 5 *S. olivacea* populations in the Sulu Sea calculated with divMigrate and based on G_ST_](#_heading=h.4d34og8).


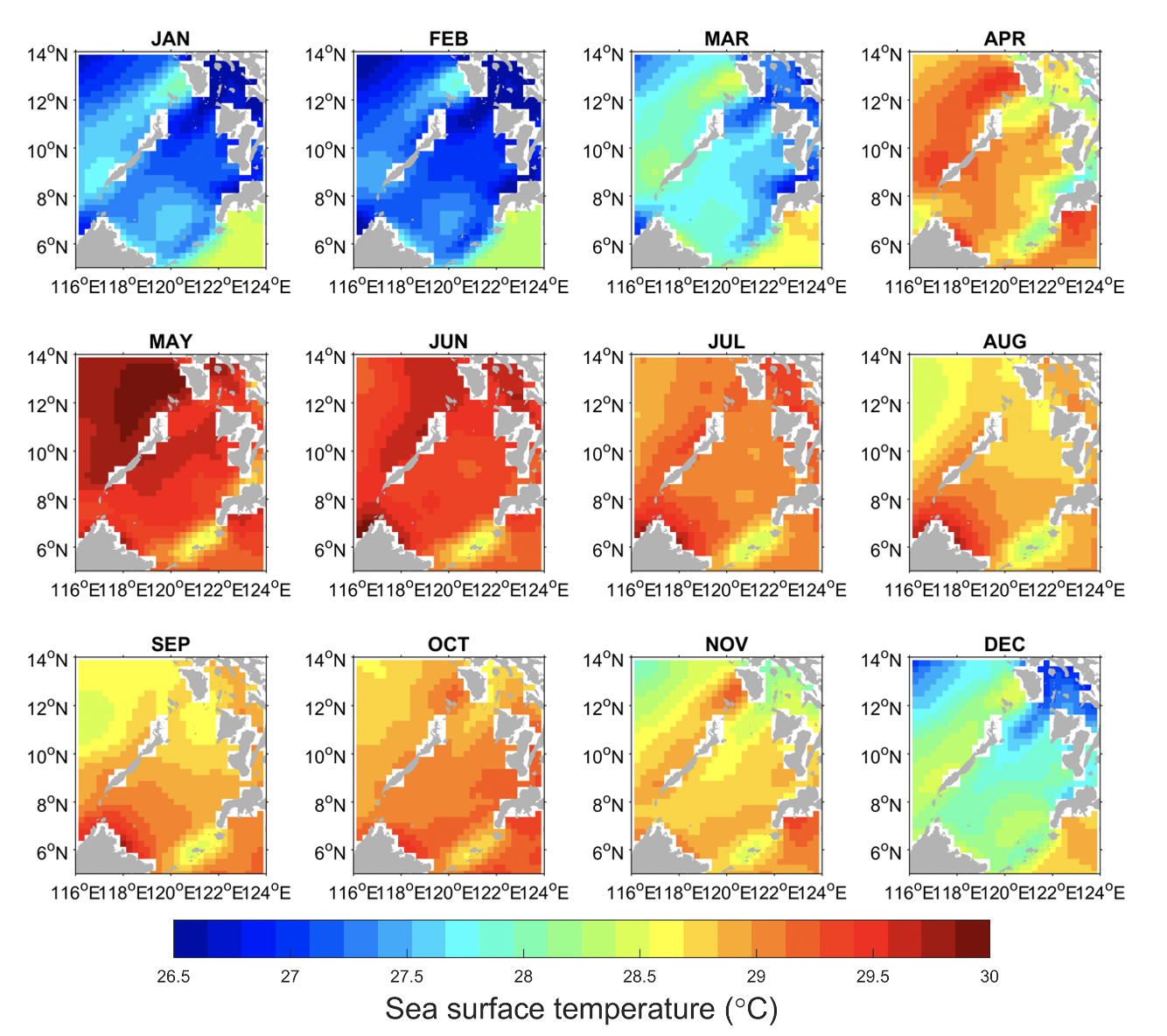


## Figure S4. Maps of average sea surface temperature (SST) in the Sulu Sea basin from January to December. Average SST was measured along 5-12**°**N and 116-124**°**E from 1987 to 2005. Data provided by the NOAA ESRL Physical Sciences Laboratory, Boulder, Colorado, USA, from their website at http://psl.noaa.gov/.
